# From fibers to cells: Fourier-based registration enables virtual Cresyl violet staining from 3D polarized light imaging

**DOI:** 10.1162/IMAG.a.1079

**Published:** 2026-01-07

**Authors:** Alexander Oberstrass, Esteban Vaca, Eric Upschulte, Meiqi Niu, Nicola Palomero-Gallagher, David Graessel, Christian Schiffer, Markus Axer, Katrin Amunts, Timo Dickscheid

**Affiliations:** Institute of Neuroscience and Medicine (INM-1), Research Centre Jülich, Jülich, Germany; Helmholtz AI, Research Centre Jülich, Jülich, Germany; Cécile & Oskar Vogt Institute of Brain Research, Medical Faculty and University Hospital Düsseldorf, Heinrich Heine University Düsseldorf, Düsseldorf, Germany; Department of Physics, University of Wuppertal, Wuppertal, Germany; Institute of Computer Science, Faculty of Mathematics and Natural Sciences, Heinrich Heine University Düsseldorf, Düsseldorf, Germany

**Keywords:** deep learning, virtual staining, fiber architecture, cytoarchitecture, polarized light imaging, Cresyl violet, vervet monkey brain

## Abstract

Comprehensive assessment of the various aspects of the brain’s microstructure requires the use of complementary imaging techniques. This includes measuring the spatial distribution of cell bodies (cytoarchitecture) and nerve fibers (myeloarchitecture). The gold standard for cytoarchitectonic analysis is light microscopic imaging of cell-body stained tissue sections. To reveal the 3D orientations of nerve fibers, 3D Polarized Light Imaging (3D-PLI) has been introduced as a reliable technique providing a resolution in the micrometer range while allowing processing of series of complete brain sections. 3D-PLI acquisition is label-free and allows subsequent staining of sections after 3D-PLI measurement. By post-staining for cell bodies, a direct link between fiber- and cytoarchitecture can potentially be established in the same section. However, inevitable distortions introduced during the staining process make a costly nonlinear and cross-modal registration necessary in order to study the detailed relationships between cells and fibers in the images. In addition, the complexity of processing histological sections for post-staining only allows for a limited number of such samples. In this work, we take advantage of deep learning methods for image-to-image translation to generate a virtual staining of 3D-PLI that is spatially aligned at the cellular level. We use a supervised setting, building on a unique dataset of brain sections, to which Cresyl violet staining has been applied after 3D-PLI measurement. To ensure high correspondence between both modalities, we address the misalignment of training data using Fourier-based registration. In this way, registration can be efficiently calculated during training for local image patches of target and predicted staining. We demonstrate that the proposed method can predict a Cresyl violet staining from 3D-PLI, resulting in a virtual staining that exhibits plausible patterns of cell organization in gray matter, with larger cell bodies being localized at their expected positions.

## 1 Introduction

To understand the organizational principles of the brain, complementary imaging techniques are used to highlight different aspects of brain architecture. Two important aspects of the microstructural organization are fiber- and cytoarchitecture (Amunts & Zilles, 2015; Nieuwenhuys, 2013). While cytoarchitecture encompasses the spatial distribution and shape of cell bodies in the cerebral cortex and subcortical nuclei, fiber architecture refers to the course and composition of nerve fibers. However, cytoarchitecture and fiber architecture are usually studied using different staining protocols, applied in different sections. Only a few protocols are available to combine cyto- and fiber staining in a single protocol, for example, Luxol fast blue (Klüver & Barrera, 1953), Bielschowsky (1904) or the triple staining by Novotny and Novotny (1977). While they allow visualizing cell bodies and fibers in one and the same section, they lack information about 3D fiber orientations. As a result, they do not support the tracing of axons and fiber bundles over long distances.

3D-Polarized Light Imaging (3D-PLI) addresses this limitation. It is a microscopic imaging technique for evaluating the three-dimensional orientation of myelinated nerve fibers in entire, unstained histological brain sections (Axer & Amunts, 2022; Axer, Amunts, et al., 2011; Axer, Graessel, et al., 2011). The technique can achieve an in-plane resolution of 1.3 µm, capturing structures at the level of individual fibers and small fiber bundles. 3D-PLI has been used to gain insights into the architecture of nerve fibers in different brain regions, such as the human hippocampus (Zeineh et al., 2017), the sagittal stratum (Caspers et al., 2022), and the vervet monkey visual system (Takemura et al., 2020). In addition, 3D-PLI has been used to validate fiber tractography algorithms and the interpretation of DW-MRI (Caspers & Axer, 2019). 3D-PLI potentially allows joint imaging of fiber tracts and neuronal cell bodies (Zeineh et al., 2017) due to diffraction patterns, differences in the density of birefringent material, and locally variable attenuation. However, this possibility has not yet been validated.

Cytoarchitecture can be studied in histological sections of postmortem brains with Cresyl violet. The staining provides contrast due to staining of the rough endoplasmic reticulum. This allows to study cell shape, density and distribution, which vary between brain regions. Due to its high spatial resolution, microscopic analysis of histological sections is considered the gold standard to verify structural parcellations (Amunts & Zilles, 2015). Recent advances in high-throughput scanning, data processing algorithms, and computational capacities have enabled the creation of 3D human brain atlases based on cytoarchitecture, such as BigBrain (Amunts et al., 2013), the Allen Adult Human Brain Atlas (AAHA, Ding et al., 2016), and the Julich Brain probabilistic atlas (Amunts et al., 2020).

Since 3D-PLI relies solely on optical properties of the tissue, it is label-free and can be combined with a staining of the same tissue after its measurement. Post-staining, for example, with Cresyl violet, enables a complementary visualization of neuronal cell bodies, potentially establishing a direct link between cytoarchitecture with 3D fiber-architecture. This requires, however, a complex histological processing, which limits the number of available samples, increases the risk of tissue damage and may lead to deformations of the section. To correct for deformation and artifacts in the images of the two modalities requires a nonlinear registration step. However, 3D-PLI and Cresyl violet stained tissue share only a few automatically identifiable cross-modal registration landmarks, such as blood vessels or morphological landmarks. Therefore, post-staining of sections imaged with 3D-PLI is feasible but technically challenging. Since it does not scale efficiently to larger datasets, it is not applicable to whole-brain stacks involving thousands of sections.

Therefore, we aim to train a deep neural network model to perform image-to-image translation from 3D-PLI to a Cresyl violet staining. Such an approach is often denoted as *virtual histological staining*, which refers to computational methods that generate color-coded images of biological tissue without the need for traditional staining techniques (Latonen et al., 2024). The methods, instead, utilize optical properties of the tissue, such as birefringence, autofluorescence, scattering, or absorption to create images that emulate the appearance of stained tissue. A virtual Cresyl violet staining spatially aligned with 3D-PLI would allow the use of established tools for cytoarchitectonic analysis, such as automatic cell instance segmentation (Upschulte et al., 2022), directly on 3D-PLI data. Furthermore, identification of cell bodies would provide detailed registration landmarks for cross-modal registration (Ounkomol et al., 2018), thereby offering the opportunity to perform joint acquisition of aligned fiber and cytoarchitecture at a larger scale than possible today. A virtual staining could, in principle, be applied to the whole brain. However, white matter regions remain challenging due to the predominance of glial cells, mainly oligodendrocytes forming the myelin sheath, which are not distinguishable from nerve fibers in the 3D-PLI signal. Therefore, in the present study, we focus our analysis on gray matter regions.

One of the earliest applications of label-free imaging for visual staining was Quantitative Phase Imaging (QPI, Curl et al., 2004). QPI measures the phase shift of light passing through a sample, producing high-resolution images that reveal the optical properties of the tissue. It was used to generate images of collagen fibers, red blood cells, and other tissue structures without staining (Curl et al., 2004; Park et al., 2018). Later, machine-learning algorithms, especially generative models, have been trained to recognize and virtually stain different tissue structures in unstained images by performing image-to-image translation. They have successfully generated color-coded images of tissue that replicate the appearance of histological stainings, such as a virtual hematoxylin and eosin (H&E), Masson’s trichrome, and Jones’ stain from QPI of label-free tissue (Rivenson et al., 2019), a transformation of H&E stained tissue into Masson’s trichrome, periodic acid-Schiff (PAS), or Jones’ stain (de Haan et al., 2021; Yang et al., 2022). However, these stains are not very good at distinguishing the different components of the nervous tissue.

Machine learning algorithms were also used to predict fluorescence-labeled images from transmitted-light z-stacks (Christiansen et al., 2018; Cross-Zamirski et al., 2022; Ounkomol et al., 2018) or 3D fluorescence structures and a FluoroMyelin stain from bright-field and polarization images of brain slices (Guo et al., 2020). The methods typically use cross-entropy (Christiansen et al., 2018), mean absolute (L1) loss (Guo et al., 2020), or style-related losses such as conditional generative adversarial network (GAN) loss (Cross-Zamirski et al., 2022; de Haan et al., 2021; Rivenson et al., 2019). While including a GAN objective encourages prediction of realistic-looking images, it has no clear mechanism to preserve content when conditioned on a particular input image, and thus may introduce artificial structures (Cohen et al., 2018). A combination with a pixel-wise reconstruction loss (e.g., L1 loss) mitigates this problem of GAN training and improves accuracy of predictions (Isola et al., 2017).

**Fig. 1.**
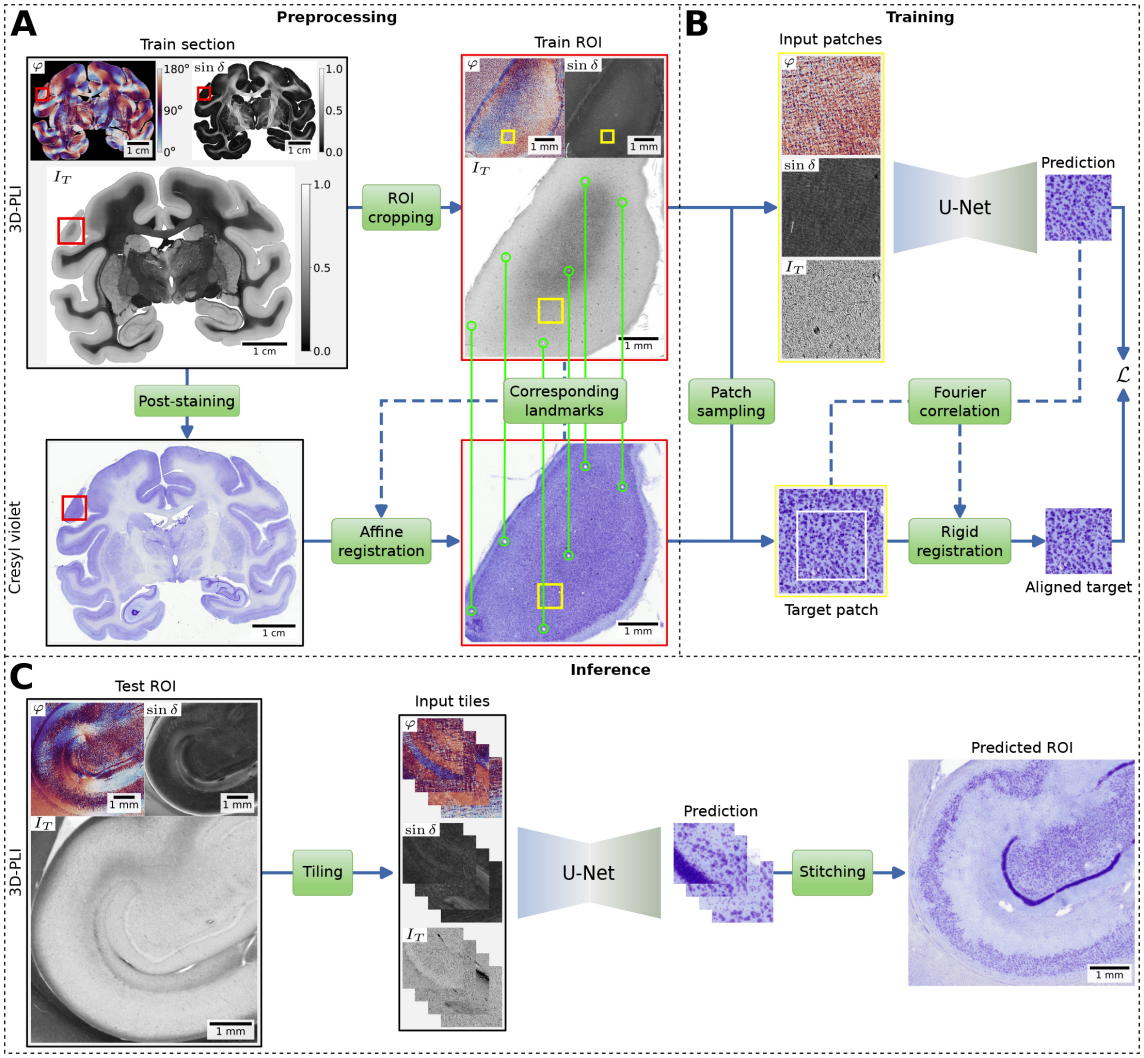
The proposed virtual staining workflow. (A) Preprocessing of 3D-PLI sections that were post-stained with Cresyl violet. As paired training data, regions of interest (ROIs) are manually cropped (red boxes) and affine registered using large blood vessels as landmarks (green marker). Background pixels in train sections are masked, and retardation values are scaled using gamma correction for visualization purposes. (B) Training of a U-Net model using patches, extracted from same random locations (yellow boxes) in 3D-PLI modalities direction φ, retardation sinδ , transmittance $I_{T}$ and the Cresyl violet staining. 3D-PLI patches are used as input to the model to predict a virtual Cresyl violet staining. The Cresyl violet patch acts as target and is rigidly aligned with the prediction during the training procedure. The alignment is performed by our proposed online registration head using Fourier-based correlation of pixels. A loss ℒ is computed between aligned target and prediction. (C) Inference using the trained U-Net model to virtually stain unseen sections or ROIs. Inputs are divided into overlapping tiles, which are processed independently by the U-Net model. The predictions are then stitched back together to form the complete virtual staining.

Since methods using paired training data for supervised image-to-image translation typically produce more accurate predictions than unpaired methods (Latonen et al., 2024; Zhu et al., 2017), a pixel-accurate alignment of training data is desired. This requires virtual staining methods to either perform a costly registration step or directly acquire paired images. A paired acquisition with 3D-PLI, however, is not feasible and a lack of structural overlap, such as a sufficient number of visible cell instances between the investigated modalities, makes pixel-accurate registration challenging. Therefore, to alleviate the need for perfectly paired training data, we propose a supervised learning objective performing local online registration of training pairs combined with a translational-invariant style comparison. This allows us to train the model on imperfectly registered image pairs with strong content preservation as in paired image-to-image translation, while enabling realistic prediction of subtle structures like cell bodies (Fig. 1).

The main contributions of our method are the following:

- We apply the matching of Gram matrix representations as a *texture sensitive style loss* for the virtual staining, as previously used for texture synthesis (Gatys et al., 2015). Since the computation of Gram matrices is translation invariant, it allows a direct comparison of image statistics between coarsely aligned training examples. It, therefore, improves the accuracy of predicted cell instances over commonly used GAN style loss (Gatys et al., 2015).
- *An online registration head* for improving registration accuracy of local image pairs after pre-alignment of larger tissue tiles during training. We consider Fourier-based registration methods, which can be computed efficiently in real-time on modern GPU hardware.
- *An equivariance loss* to improve the accuracy of cell instance predictions by addressing the inherent agnosticism of loss computation to constant displacements through online registration.

## 2 Materials and Methods

### 2.1 Microscopic imaging of histological brain sections

We demonstrate the proposed virtual staining approach on a set of brain sections for which Cresyl violet staining has been performed after 3D-PLI measurement. The brain sample for this study was obtained from a healthy 2.4-year-old adult male vervet monkey (Wake Forest-ID 1818; Axer et al., 2020; Takemura et al., 2020) in accordance with the Wake Forest Institutional Animal Care and Use Committee (IACUC #A11–219) and conforming the AVMA Guidelines for the Euthanasia of Animals. To obtain an undistorted volumetric reference, a T2 weighted MRI was acquired in-vivo 1 day prior to sacrifice. The brain was removed from the skull within 24 hours after flushing with phosphate-buffered saline and perfusion fixated with 4% buffered paraformaldehyde. It was stored for several weeks at -70°C in 20% glycerin solution for cryoprotection, and then sliced coronally with 60 µm section thickness using a large-scale cryostat microtome (Polycut CM 3500, Leica Microsystems, Germany). Blockface images of the frozen tissue block were taken with a CCD camera before cutting each brain section. The images were reconstructed into a 3D blockface volume to provide an undistorted reference for section realignment (Schober et al., 2015).

#### 2.1.1 Image acquisition

For 3D-PLI acquisition, brain sections were scanned using a polarizing microscope (LMP-1, Taorad, Germany) with 1.3 µm resolution (Axer & Amunts, 2022; Axer, Graessel, et al., 2011). The focus level of the LMP-1 was manually adjusted to the center of the tissue for each section. Inside the LMP-1 microscope, sections were placed on a specimen stage between a rotating linear and a circular polarizer on top of an incoherent light source with a wavelength of 550 ± 5 nm. Images were taken by a CCD camera for nine equidistant rotation angles ρ of the rotating linear polarizer, covering 180° of rotation. At each pixel, the measured intensity of the images followed a sinusoidal profile as

$$I_{\rho }=\frac{I_{T}}{2}(1+\text{sin}(2\rho -2\varphi )\text{sin}\delta ).$$ (1)

**Fig. 2.**
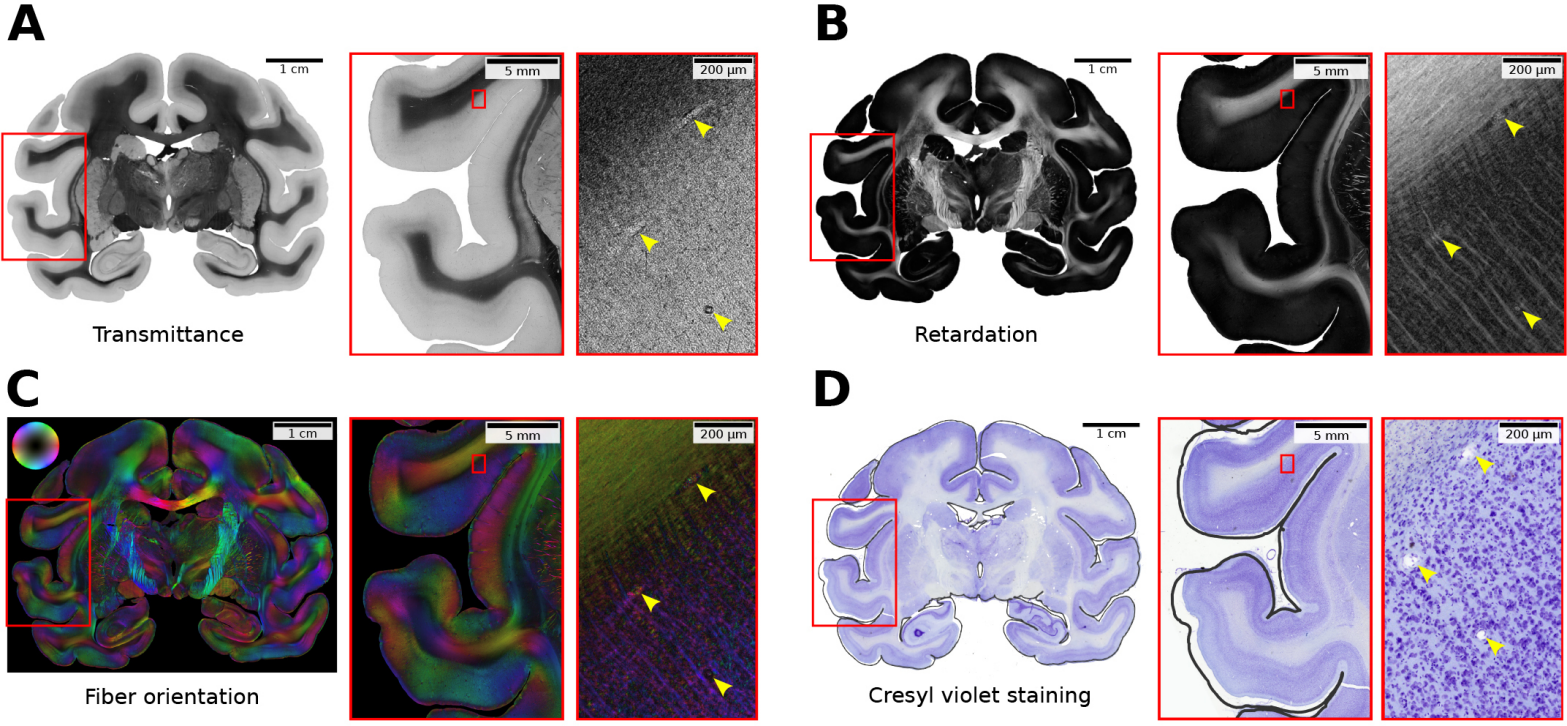
Data modalities and registration challenges for training section 544. (A–C) 3D-PLI parameter maps: Transmittance, retardation, and fiber orientation in HSV color space (hue: fiber direction; saturation/brightness: retardation). Background pixels are masked for visualization purposes only. (D) Affine registered Cresyl violet staining. The pial surface of the 3D-PLI acquisition is shown as a contour plot in D for reference. Between both data acquisitions remains a nonlinear misalignment that cannot be resolved by a global affine transformation. At a local scale, the remaining misalignment is approximately linear. Yellow arrows indicate blood vessels that can be used as mutual registration landmarks for coarse alignment.

Using harmonic Fourier analysis, parameter maps of transmittance ($I_{T}$), retardation (sinδ ), and fiber direction (φ) were obtained from the measurements, with an image size of approximately 34,000 × 44,000 pixels per section, revealing their fine-grained nerve fiber architecture (Fig. 2A–C). Each parameter map was stored in a separate HDF5 file as uncompressed 32-bit floating-point single-channel image.

After 3D-PLI acquisition, brain sections were washed, fixed and stained for cell bodies with Cresyl violet Nissl staining to reveal their cellular architecture. Whole-slide flat scans (single-plane) were performed using a Huron TissueScope LE120 high-throughput scanner at 1 µm in-plane resolution (Fig. 2D). The resulting images were saved as RGB color images with eight bit color depth (pixel values ranging from 0 to 255) in uncompressed BigTIFF format.

#### 2.1.2 Optical effects of cell bodies on the 3D-PLI signal

While 3D-PLI was primarily developed to map fiber orientations, cell bodies contribute to the measured signal as well. In the following, we summarize how absorption, diffraction, birefringence and scattering effects of cells are represented in 3D-PLI parameter maps.

Previous work reported that larger cell bodies appear as dark spots in transmittance maps (Zeineh et al., 2017), which encode light extinction caused by any material along the optical path. However, in the present transmittance maps, cell bodies are not distinguished by higher absorption relative to the surrounding fiber architecture (Fig. 2A). This is likely because their membranes remained intact due to the short postmortem time before tissue fixation.

Diffraction significantly impacts the 3D-PLI signal at the given wavelength and resolution. In cortical regions, diffraction can cause pixels of transmittance maps inside the tissue to appear brighter than the background, particularly along sharp edges such as the walls of cell bodies and blood vessels. The intensity of these diffraction patterns on the transmittance map depends on the level of the focal plane of the objective lens.

Another relevant effect is observed in retardation maps, which encode the average amount and orientation of birefringent material within each tissue voxel, primarily collagen and myelinated nerve fibers. Since cell bodies contain significantly less birefringent material than surrounding fibers, their presence causes local attenuation of the retardation appearing as dark patches between cortical fibers (Fig. 2B).

Light scattering is ubiquitous in polarized light bright-field transmission microscopy, but can generally be treated as random background noise. It does not appear to systematically affect either transmittance or retardation, with the only known exception being increased scattering at steep fibers running approximately perpendicularly to the section plane and darkening the transmittance significantly.

#### 2.1.3 Tissue shrinkage estimation

Deformations induced by histological processing include shrinkage or swelling of brain tissue. To correct these effects, the extent of shrinkage can be estimated from the ratio between the histologically processed and true brain volume, which can be represented by the fresh weight of the whole brain with an estimated mean specific density (Amunts et al., 2005, 2013) or an MRI reference (Wagstyl et al., 2020). In this work, we perform 2D segmentation of cell bodies in 3D-PLI parameter maps and measure their in-plane areas. To ensure comparability of cell sizes across studies, we estimate 2D shrinkage factors using a postmortem MRI of the same brain as a reference.

We first estimate shrinkage in the 3D-PLI acquisition by affine registration of the 3D reconstructed blockface volume to the MRI. The linear part of the affine transformation in physical space has eigenvalues [0.984, 1.005, 1.015], indicating a global volume change of less than 1% and no axis-specific systematic deviation. In a second step, we calculate 2D shrinkage factors for each brain section as the quotient between the area occupied by tissue in the 3D-PLI measurement and its corresponding blockface image. For test section #559, we estimate a global 2D shrinkage factor of 0.97, indicating a slight swelling of the 3D-PLI measurement relative to its original area within the MRI. This observation is consistent across all sections, with 2D shrinkage factors between 0.95 and 0.99. We correct the in-plane sizes of segmented cell body areas in 3D-PLI by applying the individual 2D shrinkage factor of each section, assuming an approximately uniform area change of cells and surrounding tissue.

### 2.2 Initial cross-modality alignment

After the subsequent processing of brain tissue, Cresyl violet images exhibit a deformation relative to the 3D-PLI acquisition. To align both modalities, an initial affine registration of whole brain sections is performed by manual identification of large blood vessels as landmarks visible in both modalities.

Performing the initial affine registration reveals remaining nonlinear deformations as shown in Figure 2D. Since nonlinear deformations typically have low spatial frequencies, causing smooth, large-scale distortions, we expected near-linear deformations at smaller scales. Therefore, performing an additional more local linear registration would lead to a better fit. We subsequently crop square regions of interest (ROIs) with a size of 4,096 pixels (∼5.3 mm) and without visible artifacts, covering distinct cellular architectures across the whole coronal plane. For all ROIs we perform additional affine registration and make sure that transformed landmarks have a maximum distance of 70 pixels (91 µm) from their matches (Fig. 1A). All ROIs are warped and resampled to 1.3 µm using linear interpolation to match the coordinate space of 3D-PLI. While this results in a loss of precision relative to the original resolution of Cresyl violet scans of 1 µm, matching the resolutions of both modalities facilitates subsequent processing and analysis steps.

### 2.3 Fourier-based online registration of image patches

To correct the remaining misalignment of 3D-PLI and Cresyl violet after affine registration of ROIs at a finer local scale, we introduce an online registration head that performs cross-modality alignment during training based on model predictions of small image patches (Fig. 1B). We assume that once the style transfer model has learned to reconstruct microscopic landmarks (e.g., individual cells or small blood vessels), such online registration will promote the learning of additional landmarks until the training can use pixel-aligned training examples.

The registration method performed during training needs to be computationally efficient, since training will require numerous registration iterations. Conventional feature-based image registration methods are accurate and can model nonlinear deformations but are computationally expensive and sensitive to image degradation. As we assume deformations to be approximately linear at a local scale, we take advantage of Fourier-based image correlations (Tong et al., 2019), which can efficiently recover a translation between images in the frequency domain.

#### 2.3.1 Translational shift

Fourier-based image correlation methods are able to retrieve a translational shift (Δu,Δv) between image functions f(u,v) and g(u,v) defined for integer pixel coordinates (u,v) , such that f(u,v)=g(u+Δu,v+Δv). Both functions f and g represent images of equal height H and width W and are for now assumed to repeat periodically with a periodicity of H and W, respectively.

A common approach to retrieve the translational shift between f and g is to use circular cross-correlation (Tong et al., 2019), which can be efficiently computed in the frequency domain as

$$\begin{array}{cc} \text{CC°}[a,b] & =(f⋆g)[a,b]\\ & =\sum_{u,v}f(u-a,v-b)g(u,v)\\ & =\left({ℱ}^{-1}\left\{\bar{ℱ\{f\}}ℱ\{g\}\right\}\right)[a,b], \end{array}$$ (2)

for all integer shifts [a,b] , where ℱ denotes the Fourier transformation, ${ℱ}^{-1}$ its inverse, $\bar{ℱ\{f\}}$ its complex-conjugate Fourier coefficients, and where we sum over all pixel coordinates (u,v) . The translational shift can subsequently be recovered by the location of the maximum value in CC° as

$$(\Delta u,\Delta v)=\underset{\left(a,b\right)}{\text{argmax}}\text{CC°}[a,b].$$ (3)

Eq. (2) can be extended to the mean over squared distances between pixel values as

$$\begin{array}{cc} \text{MSE°}[a,b] & =\frac{1}{HW}\sum_{u,v}{\left(f(u-a,v-b)-g(u,v)\right)}^{2}\\ & =\frac{-2(f⋆g)[a,b]+\sum_{u,v}(f^{2}(u,v)+g^{2}(u,v))}{HW}. \end{array}$$ (4)

The translational shift (Δu,Δv) can be recovered analog to Eq. (3) by computing the argmin . For periodic image functions f and g, solutions of CC° and MSE° are identical as the sum over squared functions $f^{2}$ and $g^{2}[0,1]$ is constant (Fienup, 1997).

Since histological images are not periodic, zero padding is applied to fill both images up to a shape of $\left(H_{f}+H_{g}-1,\;\;W_{f}+W_{g}-1\right)$ , in order to break periodicity and allow processing of images with different heights $H_{f}$, $H_{g}$ and widths $W_{f}$, $W_{g}$. The zero-padded images are denoted by new image functions $f_{0}$ and $g_{0}$. Furthermore, additional masks $M_{f}$ and $M_{g}$ are introduced, which have a value of one at all pixel coordinates within original height and width and zero elsewhere. We reformulate Eq. (4) to a non-circular form as

$$\text{MSE}\left[a,b\right]=\left(\frac{\left(f_{0}^{2}⋆M_{g}\right)-2\left(f_{0}⋆g_{0}\right)+\left(M_{f}⋆g_{0}^{2}\right)}{M_{f}⋆M_{g}}\right)\left[a,b\right],$$ (5)

which can be efficiently computed by multiple applications of Eq. (2). Here, the cross-correlations of $f_{0}^{2}$ and $g_{0}^{2}$ with masks $M_{g}$ and $M_{f}$, respectively, ensure that pixel values of the original unpadded images are not compared with zero padding values. Furthermore, the score is divided by the correlation between $M_{f}$ and $M_{g}$ to account for the number of overlapping pixels between original unpadded images. We apply the same division by $M_{f}*M_{g}$ also for CC° to retrieve a non-circular variant called CC. While the solutions for circular CC° and MSE° are identical, the solutions for non-circular CC and MSE differ, resulting in distinct registration metrics.

#### 2.3.2 Rotation and scale

While relative scale and rotation between images can also be retrieved in the frequency domain (Reddy & Chatterji, 1996; Sheng, 1989), we expect only small relative rotation angles and minor scale variations due to the initial affine registration by matching blood vessels. Therefore, we leverage the parallel processing capability of GPUs to perform an exhaustive search over a fixed set of rotation angles without scaling adjustments. We calculate registration metrics for every translational shift and rotation and select the rotation angle and shift combination that yields a global optimum.

### 2.4 Conditional generation of Cresyl violet staining

**Fig. 3.**
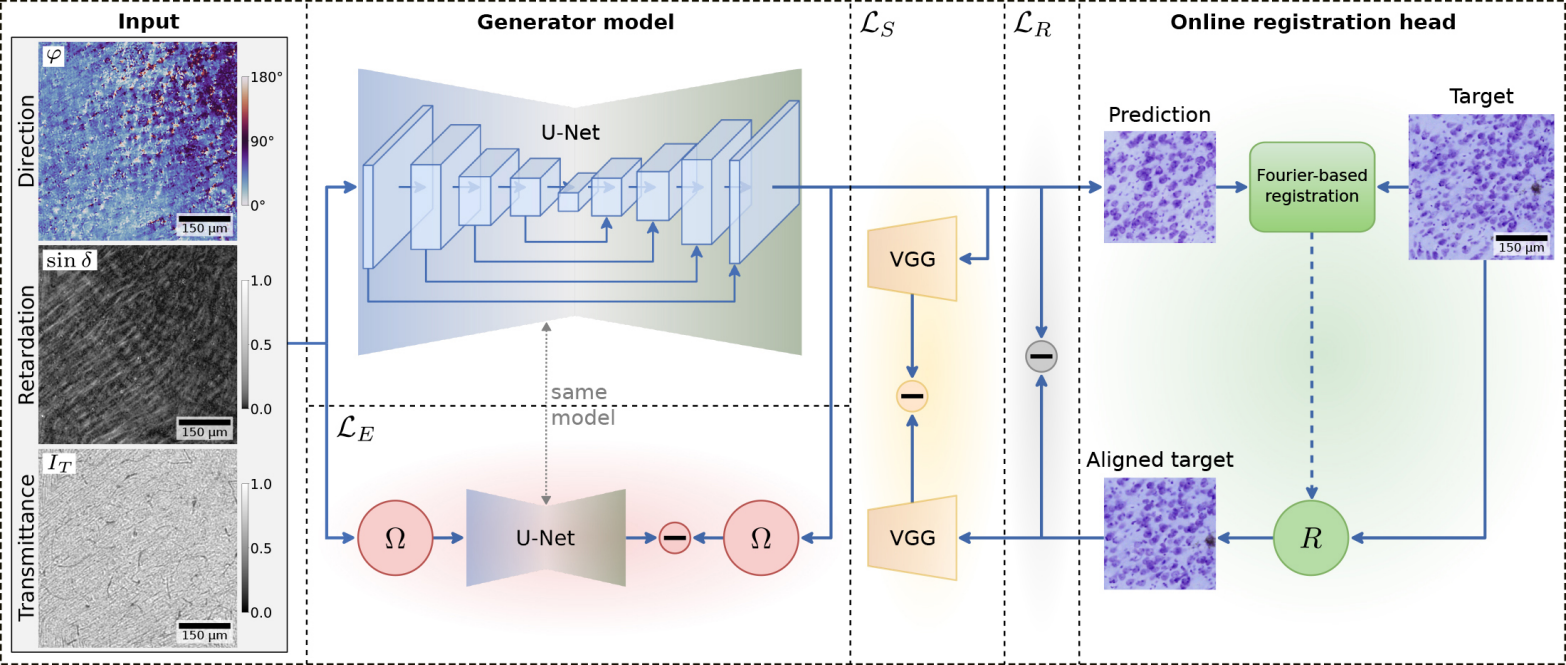
Illustration of the proposed virtual staining approach. Patches of 3D-PLI parameter maps transmittance $I_{T}$, retardation sinδ (scaled using gamma correction for visualization), and direction φ are used as input to a 2D convolutional U-Net model as generator to predict a virtual Cresyl violet staining. An online registration head estimates a rigid transformation R between a coarsely aligned Cresyl violet target patch and the prediction via Fourier-based registration. Transformation R is used to align target and prediction at the patch level. We calculate three distinct loss components: ${ℒ}_{R}$, ${ℒ}_{S}$ and ${ℒ}_{E}$. Reconstruction loss ${ℒ}_{R}$ performs a pixel-wise comparison between prediction and aligned target. Style loss ${ℒ}_{S}$ compares feature maps of a VGG network encoder using Gram matrices to mimic the style of the target image. Equivariance loss ${ℒ}_{E}$ applies the same U-Net model a second time to a rotated version of the input by rotation Ω. The output is compared with the prediction rotated by same rotation Ω, which promotes stability and avoids learning a constant shift of pixels in the prediction.

Image-to-image translation refers to the process of generating images using a generator model G conditioned on an input image x. Predictions G(x) of the model are compared with actual target images y. In our case, we translate from the domain of 3D-PLI images to Cresyl violet. The translation is performed by a U-Net (Ronneberger et al., 2015) serving as the generator model, which forms the core of our image-to-image translation framework, as illustrated in Figure 3. The three loss components used to train the U-Net are described below.

#### 2.4.1 Reconstruction loss

Similar to Isola et al. (2017), we use an $L_{1}$ loss between target y and prediction G(x) to encourage pixel correspondence in the *reconstruction loss*

$${ℒ}_{R}=E_{x,y}\left[||R_{y,G\left(x\right)}\left(y\right)-G\left(x\right)|{|}_{1}\right],$$ (6)

where $R_{y,G\left(x\right)}$ performs the proposed online registration to spatially align y and G(x) before loss calculation. This enables utilization of imperfectly aligned training data to penalize any discrepancies in corresponding pixel values between G(x) and y. To disable online registration, $R_{y,G\left(x\right)}$ can be replaced by the identity function.

#### 2.4.2 Style loss

We consider two alternative implementations of a style loss ${ℒ}_{S}$, both focussing on style preservation. We refer to the first one as *Gram loss*, and the second as *GAN loss*.

For the Gram loss, we apply a texture-sensitive style loss proposed by Gatys et al. (2015) based on squared distances between Gram matrix representations of neural network features. Given a pre-trained VGG encoder (Simonyan & Zisserman, 2015), Gram matrix representations are computed from feature activations of its layers to characterize the texture of images at different complexities. For each layer l, the encoder produces a different number of $N_{l}$ feature maps, each storing $K_{l}$ spatial entries (i.e., height × width). Elements of the Gram matrix $\Gamma_{ij}^{l}$ at layer l are computed as the inner product between the i-th and j-th feature map $F_{i}^{l}$ and $F_{j}^{l}$, where each map is flattened to a $K_{l}$-dimensional vector:

$$\Gamma_{ij}^{l}=\sum_{k=1}^{K_{l}}F_{ik}^{l}F_{jk}^{l}.$$ (7)

Since the Gram matrix computation captures global feature correlations rather than spatial locations, this allows a translation-invariant comparison of image statistics. The style loss is computed over all L layers of the VGG encoder, using Gram matrix representations $\Gamma^{l}$ for the online registered target and ${\hat{\Gamma }}^{l}$ for the prediction:

$${ℒ}_{S}=E_{x,y}\left[\sum_{l=1}^{L}\frac{1}{K_{l}^{2}N_{l}^{2}}\sum_{i=1}^{N_{l}}\sum_{j=1}^{N_{l}}{\left(\Gamma_{ij}^{l}-{\hat{\Gamma }}_{ij}^{l}\right)}^{2}\right].$$ (8)

For the GAN loss, we consider adversarial training (Goodfellow et al., 2014) to compare with previous work in virtual staining (Cross-Zamirski et al., 2022; Rivenson et al., 2019). We implement the GAN loss in the form of a Wasserstein GAN (Arjovsky et al., 2017). In contrast to conditional GAN training (Isola et al., 2017; Mirza & Osindero, 2014), we do not condition the discriminator on input images x, as this would cause the model to reproduce any misalignment in the training data.

#### 2.4.3 Equivariance loss

By registering target y to generator prediction G(x) before loss calculation, displacements of objects in G(x) relative to x are not captured by ${ℒ}_{R}$. To prevent pixel shifts in G(x), we enforce equivariance with respect to rotations through the *equivariance loss*

$${ℒ}_{E}=E_{x}\left[{\left\|\text{Ω}\left(G\left(x\right)\right)-G\left(\text{Ω}\left(x\right)\right)\right\|}_{2}\right],$$ (9)

where operator Ω represents an image rotation of 180° . Minimizing Eq. (9) ensures that pixels in G(x) correspond to pixels at the same pixel coordinates in x as any discrepancy would cause a mismatch of Ω(G(x)) and G(Ω(x)).

#### 2.4.4 Total loss formulation

All components are aggregated into the *total loss*

$$ℒ=\lambda {ℒ}_{R}+\left(1-\lambda \right){ℒ}_{S}+\eta {ℒ}_{E},$$ (10)

with relative weightings λ∈[0,1] and η≥0 as hyperparameters. We denote models that use Gram loss as the style loss ${ℒ}_{S}$ as *Gram*, and models that use GAN loss as the style loss ${ℒ}_{S}$ as *GAN*. When online registration is enabled during computation of reconstruction loss ${ℒ}_{R}$, the corresponding models are denoted as *Gram+Reg* and *GAN+Reg*. Base models Gram and GAN compute the reconstruction loss with online registration disabled.

### 2.5 Model training

For the generator G, we use the same 5-layer U-Net (Ronneberger et al., 2015) with numbers of features [32, 64, 128, 256, 512] in all experiments, and adjust the input and output channels to 3 according to our setup. To train G, we use square 3D-PLI patches of 444 pixels size, represented by parameter maps transmittance ($I_{T}$), retardation (sinδ ) and direction (φ). We reformulate the 3D-PLI parameters as triplets $\left(I_{T},\;\text{sin(}\delta \text{)}\;\text{cos(}2\varphi \text{)},\;\text{sin(}\delta \text{)}\;\text{sin(}2\varphi \text{)}\right)$ to resolve the circular behavior of direction φ, standardize the channels and stack them to the input of generator G. Due to the fully convolutional approach of the U-Net model without padding, the generated output predictions have a reduced size of 260 pixels. Unless specified otherwise, we use a patch size of 360 pixels for the target Cresyl violet images, centered at the input patch position. They are chosen to be larger than the model predictions to allow the online registration to correct translational shifts of up to 50 pixels in any direction, while keeping the predictions fully contained within the target images. We normalize image pixel values of the Cresyl violet staining to the range of [0, 1].

For computing style loss ${ℒ}_{S}$, we extract features from a VGG19 model (Simonyan & Zisserman, 2015) to compute the *Gram loss*. The VGG feature encoder network has a depth of four layers and three input channels with pre-trained weights on ImageNet (Deng et al., 2009). We multiply the style loss ${ℒ}_{S}$ by a constant factor of 10^4^ to bring it to the same order of magnitude as reconstruction loss ${ℒ}_{R}$. For the training, we use Adam optimizer (Kingma & Ba, 2017) with $\beta_{1}$ = 0.9, $\beta_{2}$ = 0.999 and a learning rate of 10^-3^. If not stated otherwise, we use η = 0.1 and λ = 0.5 as default in the training objective in Eq. (10).

In the case of *GAN* style loss ${ℒ}_{S}$, we use a 4-layer convolutional network as discriminator with kernel size 4, stride 2, padding 1, and feature size of [32, 64, 128, 256], followed by batch normalization (Ioffe & Szegedy, 2015) and Leaky ReLU after each convolution. For training, we use the Wasserstein GAN (Arjovsky et al., 2017) objective and a separate Adam optimizer for the discriminator and the generator, using $\beta_{1}$ = 0.5, $\beta_{2}$ = 0.999 and a learning rate of 10^-4^. We perform five updates for the discriminator for one update of the generator and clamp discriminator weights at 0.03 after each step.

#### 2.5.1 Training data

**Fig. 4.**
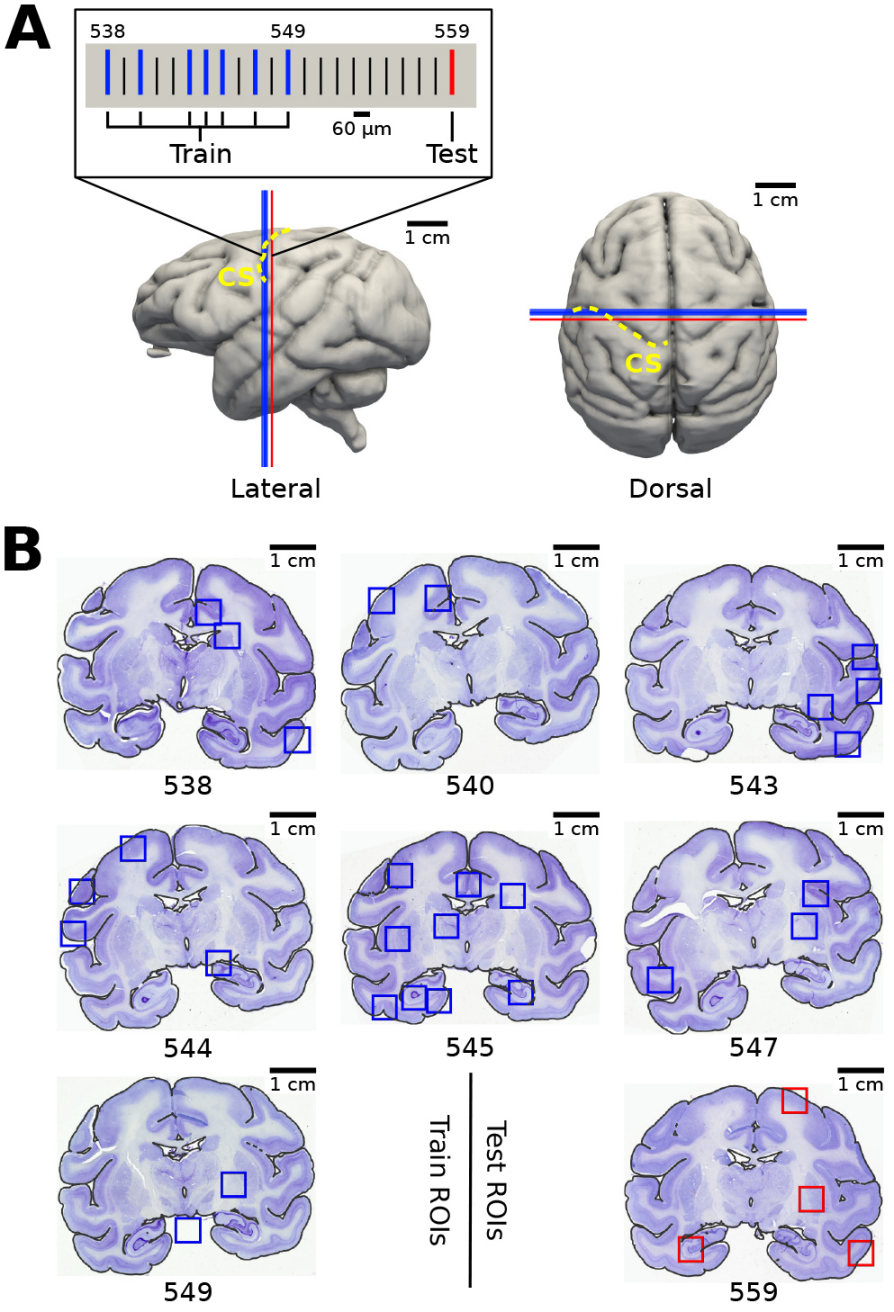
Localization of train and test data. (A) Seven sections used for training (blue stripes) and one section used for testing (red stripe) were taken at the level of the central sulcus (CS; yellow dashed lines). Locations are shown on top of the 3D reconstructed blockface of the brain for reference. Train and test data are 0.6 mm apart from each other. (B) Selected locations of train and test regions of interest (ROIs), which are used for training and testing the models. The images show ROIs from each of the train and test sections on top of globally affine registered Cresyl violet images. Black contour plots outline the pial surface of corresponding 3D-PLI sections for reference.

For model training and evaluation, we use eight coronal sections at the level of the central sulcus. Seven sections are used for training and one section is kept for testing with a gap of 0.6 mm between train and test sections (Fig. 4A). From the training sections, 27 affine-aligned ROIs are extracted (Fig. 4B), where one ROI is held out for validation to identify possible overfitting. For each ROI, we extract joint target Cresyl violet images and 3D-PLI modalities at the same center location. To maximize the diversity of the training examples, we do not pre-compute training patches but sample them randomly during the training process. We use a batch size of 128 and draw 32,768 paired random patches per epoch evenly distributed across the training ROIs, resulting in approximately 1,260 random samples per ROI per epoch. All training is performed for 150 epochs or until model convergence if validation loss did not decrease for at least 50 epochs.

#### 2.5.2 Data augmentation

To enhance the robustness of our trained models we employ 3D-PLI-specific data augmentations, which were carefully modified to keep physically plausible signal parameters (Oberstrass et al., 2024). Specifically, we perform random rotation by angles between -180° and 180° with mirror padding and horizontal and vertical random flipping. In both cases, direction parameter maps φ are corrected accordingly. Additionally, we perform Gaussian blurring of 3D-PLI parameter maps for random standard deviations up to σ = 1.5 and kernel sizes of 3 or 5. We scale thickness and attenuation coefficients for 3D-PLI parameter maps by random values between 0.5 and 2.

#### 2.5.3 Implementation

All models were trained on the supercomputer JURECA-DC at the Jülich Supercomputing Centre (JSC, Thörnig, 2021) on a single node by splitting each batch equally onto 4 NVIDIA A100 GPUs using distributed data-parallel strategy. For data pre-processing 128 worker processes were spawned on 128 CPU cores. For reference, training for 100 epochs took 8 hours with online registration and 4 hours without on this hardware. The implementations are based on the Quicksetup-ai template by the HelmholtzAI Consultants Munich,^1^ using the frameworks: *PyTorch* (Paszke et al., 2019), *PyTorch Lightning* (Borovec et al., 2022) and *Hydra* (Yadan, 2019).

#### 2.5.4 Online registration

For the online registration head, we restrict accepted solutions of Eq. (5) to translation + rotation pairs that cause the registered target to have full overlap with the prediction, avoiding loss calculation over zero-padded values. We check the translation correction for 31 rotation angles from -7.5° to 7.5° with steps of 0.5° and take the translation + rotation pair with the best registration score.

## 3 Experiments and Results

We compare a selection of performance scores to identify optimal hyperparameters of the proposed method and assess the overall potential of the best performing model. The main hyperparameters are the choice of the online registration metric, the type of style loss ${ℒ}_{S}$, its relative weighting λ to reconstruction loss ${ℒ}_{R}$, and whether to use the additional equivariance loss ${ℒ}_{E}$. To reduce the massive computational demands by a rigorous grid search across all hyperparameters, we choose to identify a suitable choice for the online registration metric and model variants using different loss components independently before determining an optimal relative weighting λ.

### 3.1 Experiment setup

#### 3.1.1 Test data

**Fig. 5.**
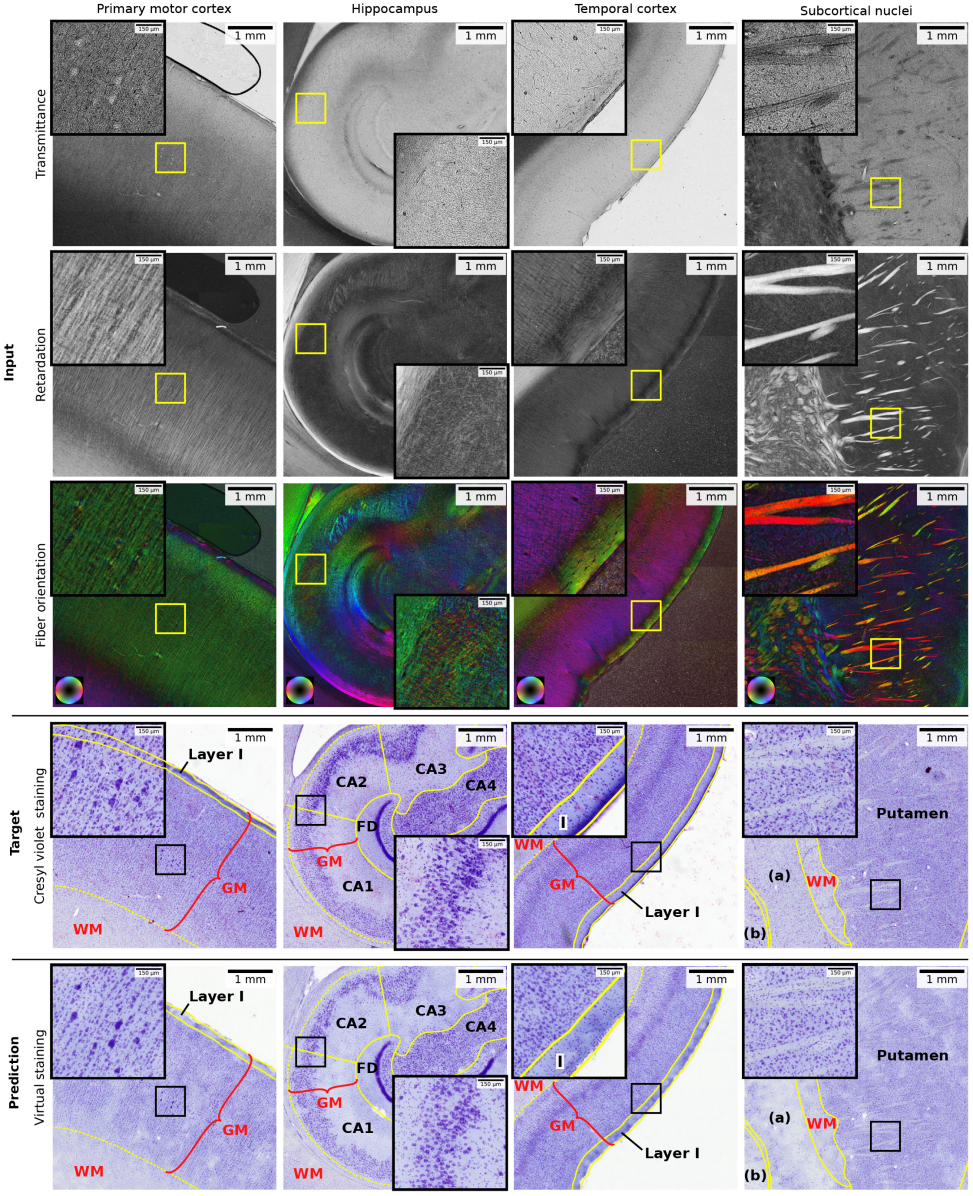
Overview of ROIs used for the evaluation, which represent distinct cellular architectures. They were extracted from section 559, located 0.6 mm apart from the training sections. Embedded windows show magnified details inside each ROI. Columns each show one of the four test ROIs taken from the anterior subdivision of the primary motor cortex (4a), the hippocampal cornu Ammonis (CA) region, temporal cortical area TE, and parts of the putamen and globus pallidus (GP; a: GP Pars interna; b: GP Pars externa) as subcortical nuclei. The first three rows demonstrate 3D-PLI modalities transmittance, retardation (scaled using gamma correction for visualization) and fiber orientation in HSV color space (hue: fiber direction; saturation/brightness: retardation). The 3D-PLI modalities are compared to the registered target Cresyl violet and predicted virtual staining.

We manually select four ROIs for model evaluation, ensuring a diverse representation of different cytoarchitectonic characteristics from the held-out test section (Fig. 4B). The ROIs contain primary motor cortex 4a, temporal cortical area TE, the hippocampal cornu Ammonis (CA) region, and parts of the putamen and globus pallidus (Pars interna and Pars externa) as subcortical structures (Fig. 5).

As the computation of image metrics requires a precise alignment of test data, we perform elastic registration of test ROIs based on landmarks and image intensity using the *bUnwarpJ* (Arganda-Carreras et al., 2006) algorithm. We use predictions of an independently trained Gram+Reg model as target. The predictions are used to manually identify 15–25 characteristic cell clusters as landmarks per ROI, which are confirmed by the location and arrangement of faint shadows of cells visible in the 3D-PLI transmittance. For registration, we use an image weight of 1.0, a landmark weight of 10.0, and a consistency weight of 10.0. The strong weights for landmarks and consistency are chosen to prevent the transformation field from overly conforming to the predictions and to overcome local optima.

#### 3.1.2 Evaluation scores

We evaluate the impact of different model parameter choices on the quality of the predicted virtual staining by applying structural similarity index measure (SSIM, Wang et al., 2004), mutual information (MI), and root-mean-square error (RMSE). For each metric, we report the mean over all test ROIs. To evaluate how well cell positions are preserved by different models, we compute F1 scores based on cell instance segmentations by a contour proposal network (CPN, Upschulte et al., 2022, 2023). Predicted cell instances are obtained from segmentations of the virtual staining and compared to target cell instances from the corresponding Cresyl violet images. For each ROI, predicted and target cells are matched by calculating their intersection over union (IoU), requiring a minimum IoU of 30% for a match. Each target cell can be a match for at most one predicted cell. Matched instances are counted as true positives, unmatched predicted cells as false positives, and unmatched target cells as false negatives. F1 scores are then computed from aggregated counts across all ROIs. For the cell detection model, we fine-tuned a pre-trained CPN^2^ for cell body segmentation in cell-stained microscopy images using the *celldetection*^3^ Python package. Fine-tuning was performed on a diverse mix of manually annotated images, as well as synthetic data.

We restrict the computation of evaluation metrics to gray matter, where most of the neuronal cell bodies are located, excluding white matter and background pixels. In test ROIs showing cortical regions, we further exclude molecular layer I as it consists of only a few individual neurons and exhibits a nonlinear deformation due to tissue shrinkage, which could not be reliably corrected in the registration. For the ROI showing the hippocampus, we exclude the fascia dentata, as its granular layer consists of very densely packed neurons, which cannot reliably be distinguished and restrict the analysis to hippocampal CA1-CA4 regions.

### 3.2 Online registration metric

To compute a pixel-aligned reconstruction loss we apply the online registration head (Section 2.3). We consider correlation-based registration metrics CC and MSE with phase correlation (PC, Kuglin, 1975) and blur-invariant phase correlation (BIPC, Ojansivu & Heikkila, 2007). We apply a Hann window to PC and BIPC before their calculation to mitigate their bias towards the sharp image edges (Gonzales & Woods, 2008).

To understand how image degradation effects can influence the success of the online registration head, we compare the robustness of the metrics against noise and blur. A square target image with 460 pixels size is extracted from a random location in the Cresyl violet staining. Next, a smaller moving tile with a size of 260 pixels is extracted from a random location within the target and distorted by gradually increasing noise or blur on the image. We add noise from a zero-centered Gaussian distribution with standard deviation σ increasing from 0 to 25 and Gaussian blur with increasing kernel sizes with standard deviations σ growing from 0 to 50. The registration head is applied with each metric to realign the degraded moving tile with its location in the target image. We compare the hit rate over 100 examples per degradation step. The registration is considered successful if the determined translational displacement remains within 5 pixels of the actual displacement.

**Fig. 6.**
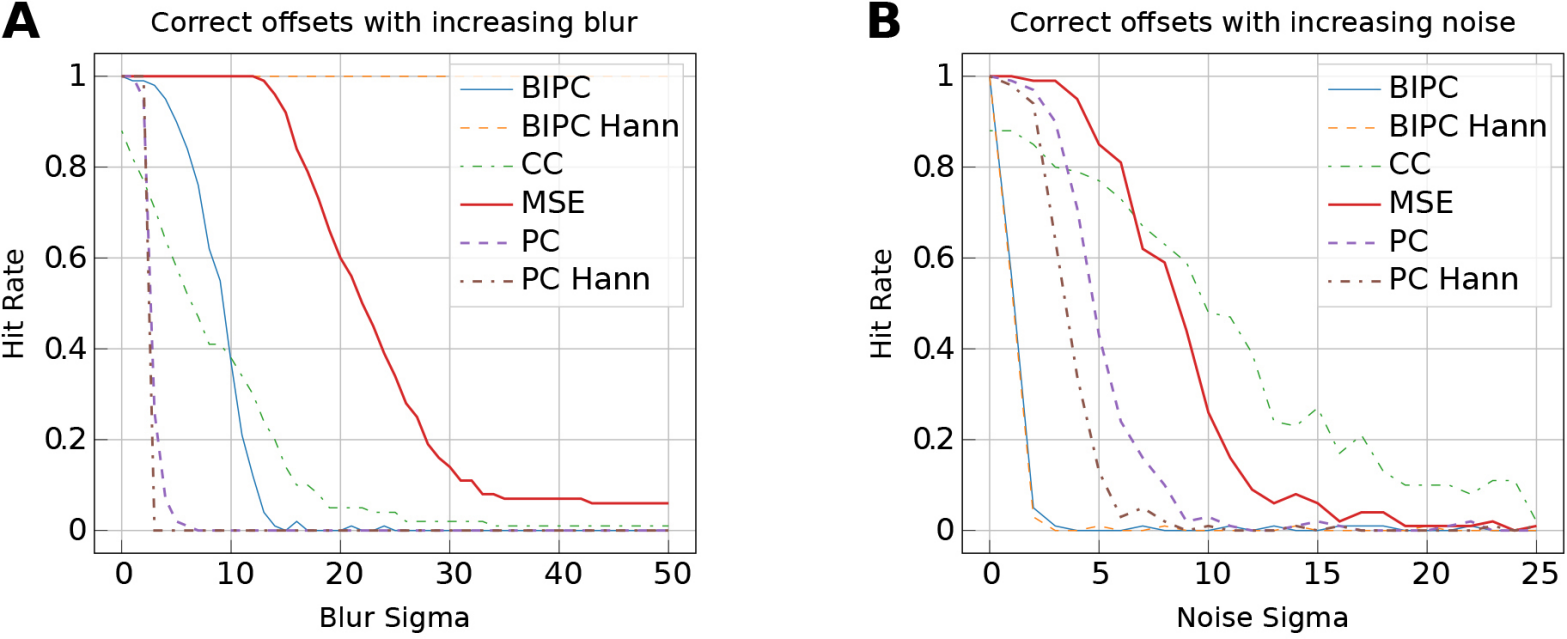
Robustness of different online registration metrics against synthetic image distortions. We report the proportion of correct rigid alignments for different blur (A) and noise (B) levels by registration metrics MSE, CC, PC, and BIPC. The registration is considered successful (a hit) if the translational displacement obtained remains within 5 pixels of the actual displacement.

Figure 6 shows that as blur increases, BIPC with a Hann window achieves the best performance. This result is expected, as the metric remains invariant to blur. We also observe that CC and MSE perform best in mitigating the effects of noise, while the other metrics fail already with a minor amount of noise. Overall, MSE provides a balanced tradeoff between blur and noise response.

To make an optimal choice of a registration metric for online registration, we train Gram+Reg models with CC, BIPC, PC, and MSE compared to a Gram model without online registration. We use a Cresyl violet target patch size of 360 pixels and predictions of 260 pixels, centered within the target patch. This allows the online registration to correct for a maximum of 50 pixels translation in each dimension. For Gram+Reg models trained with CC, BIPC and the Gram model, λ=0.1 was chosen as it improved results compared to λ=0.5 for PC and MSE.

Table 1 shows a quantitative comparison of the models. Independent of the metric, the best results are observed when using Gram+Reg with online registration Among all metrics considered, the model with MSE performs best.

**Table 1.** Performance of models trained with different online registration metrics in terms of similarity of generated images with ground truth.

Method	Metric	MI ↑	RMSE ↓	SSIM ↑	F1 ↑
Gram	-	0.126	33.6	0.354	30.2
Gram+Reg	CC	0.136	33.0	0.379	34.4
	BIPC	0.149	31.5	0.371	32.6
	PC	0.185	31.9	0.415	38.0
	MSE	**0.226**	**29.8**	**0.444**	**41.3**

We compare cross-correlation (CC), phase-correlation (PC), blur-invariant phase-correlation (BIPC), and mean-squared error (MSE) as online registration metrics against using no online registration (-). Arrows indicate the direction of better performance (↑ higher is better, ↓ lower is better). Best scores per column in bold.

### 3.3 Performance analysis of different model variants

**Fig. 7.**
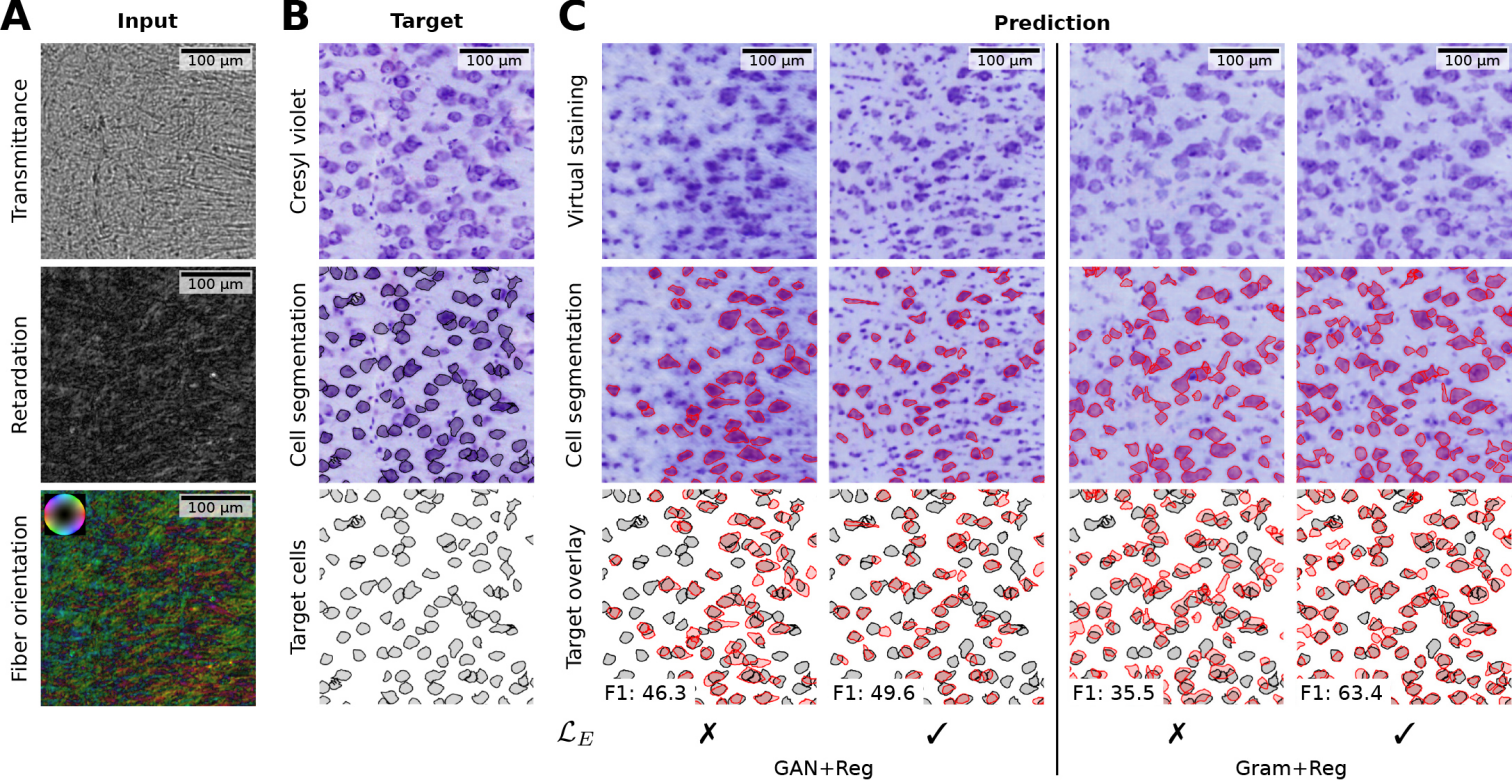
Example image illustrating how equivariance loss ${ℒ}_{E}$ improves cell instance overlap of Gram+Reg and GAN+Reg models. (A) 3D-PLI input as transmittance, retardation (scaled using gamma correction) and fiber orientation map in HSV color space (hue: fiber direction; saturation/brightness: retardation). (B) Registered target Cresyl violet staining, a cell segmentation by a CPN model, and the identified target cell instances. (C) Predicted virtual staining of models trained with equivariance loss ${ℒ}_{E}$ enabled (✓) or disabled (✗). Predicted cell instances (red) by each model are overlaid with the target cells (gray) and F1 scores computed for cells in this specific image patch. Since the displacement occurs randomly, a patch from our test set and random seeds for the models were manually selected that illustrate that effect. The patch shows the pyramidal layer of the hippocampal CA1 region.

To justify including equivariance loss ${ℒ}_{E}$ (Eq. (9)) in the total loss, Figure 7 illustrates the effect of training different models with this loss component enabled and disabled. With ${ℒ}_{E}$ disabled (η = 0), cell instances in the model predictions exhibit a relative displacement. Enabling ${ℒ}_{E}$ (η = 0.1) improves the overlap of cell instances with the original Cresyl violet staining.

To quantify the impact of the loss component ${ℒ}_{E}$ on the predictions, we compare the models with ${ℒ}_{E}$ enabled and disabled in Table 2. We consider Gram and Gram+Reg, as well as GAN and GAN+Reg as model variants. All models use λ=0.5 expect for the Gram model without online registration, where λ=0.1 showed better performance. Since displacements of cell instances in the model predictions occur randomly with varying strength, we report mean and standard errors across four independent trainings. Models trained with ${ℒ}_{E}$ enabled show the highest correspondence with the target staining. Without online registration, the advantage of including ${ℒ}_{E}$ becomes negligible. Furthermore, results in Table 2 demonstrate that Gram models outperform their corresponding GAN models in all evaluation metrics. Regardless of the variant used for style loss ${ℒ}_{S}$, all models benefit from the online registration. We present averaged scores across all test ROIs. Individual scores per ROI, are provided in Appendix Table A1 as additional information.

**Table 2.** Effect of the proposed online registration on models trained with different choices of style loss ${ℒ}_{S}$.

${ℒ}_{S}$	Reg.	${ℒ}_{E}$	MI ↑	RMSE ↓	SSIM ↑	F1 ↑
Gram	✓	✓	**0.224** ± 0.002	**29.6** ± 0.4	**0.445** ± 0.002	**41.0** ± 0.4
		✗	0.211 ± 0.004	30.2 ± 0.4	0.432 ± 0.004	35.2 ± 1.2
	✗	✓	0.111 ± 0.012	33.6 ± 0.8	0.314 ± 0.023	22.6 ± 4.8
		✗	0.107 ± 0.007	34.2 ± 0.3	0.304 ± 0.015	22.5 ± 3.1
GAN	✓	✓	0.160 ± 0.008	30.5 ± 0.5	0.389 ± 0.012	34.7 ± 1.6
		✗	0.126 ± 0.008	33.0 ± 0.4	0.347 ± 0.010	28.0 ± 1.3
	✗	✓	0.085 ± 0.008	38.1 ± 0.5	0.249 ± 0.001	12.9 ± 0.3
		✗	0.096 ± 0.008	38.8 ± 1.7	0.253 ± 0.005	13.9 ± 1.5

Each variant is trained with online registration head (Reg.) and equivariance loss (${ℒ}_{E}$) enabled or disabled. MSE metric is used for online registration and η = 0.1 for weighting of ${ℒ}_{E}$. The deviation is reported as standard error over four independent trainings with different random seeds. Arrows indicate the direction of better performance (↑ higher is better, ↓ lower is better). Best scores per column in bold.

**Fig. 8.**
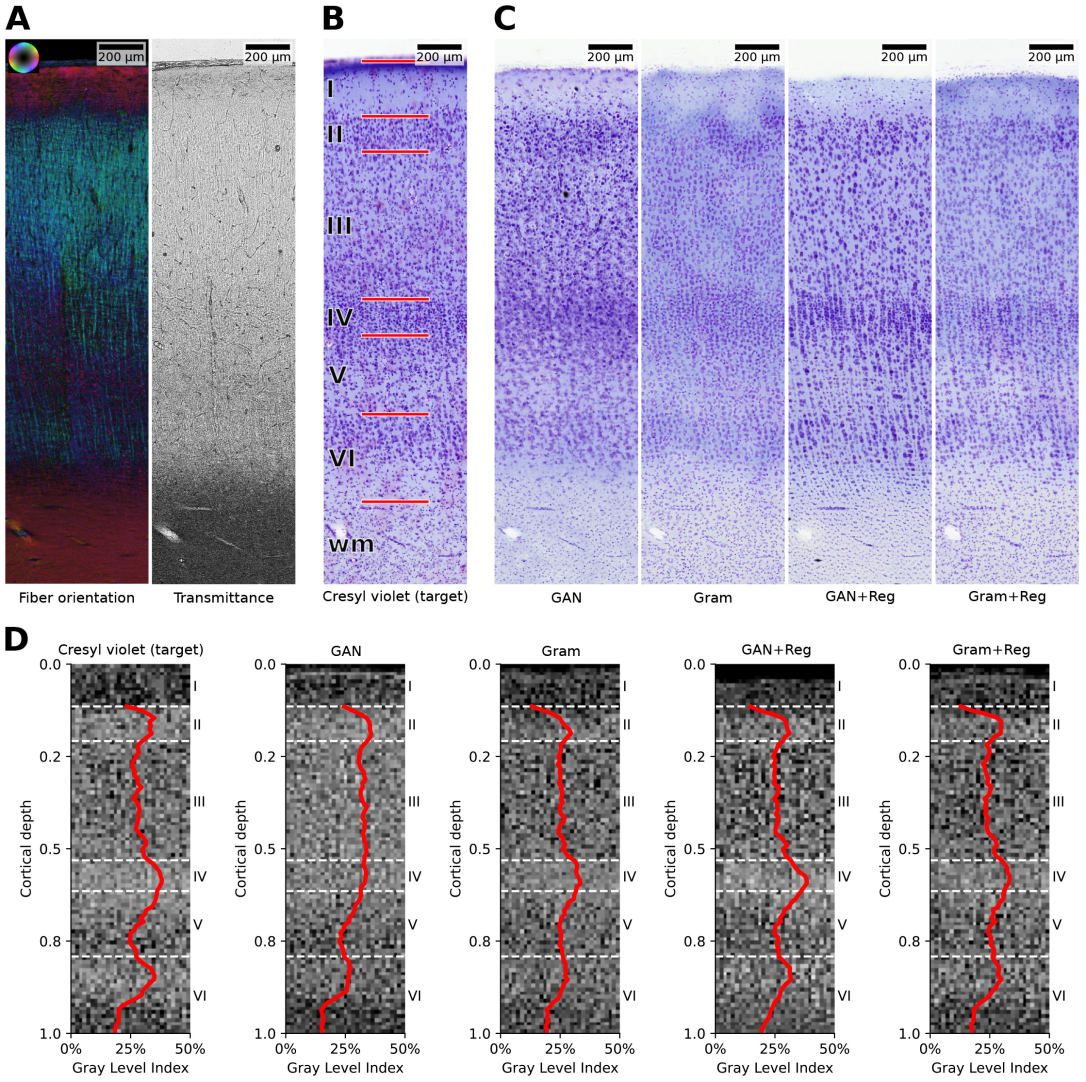
Comparison of virtual stainings for a patch of temporal area TE. (A) 3D-PLI input visualized as fiber orientation in HSV color space (hue: fiber direction; saturation/brightness: retardation) and transmittance. (B) Registered target Cresyl violet staining for reference with annotations of cortical layers I–VI and white matter (wm). (C) Predicted virtual stainings of GAN and Gram models and extended variants using MSE online registration (GAN+Reg, Gram+Reg). (D) GLI images ranging from the pial surface to the cortex/white matter transition and average profiles (red lines) for layers II–VI.

A comparison of model predictions is shown in Figure 8C for the whole cortical depth of an Isocortex sample from temporal cortical area TE. GAN+Reg and Gram+Reg provide the most realistic-looking reconstructions in terms of relative size and shape of cell bodies, along with the differences between layers concerning cell packing density. While Gram+Reg seems to be influenced by technically-related inhomogeneities in staining intensity, GAN+Reg produces a clearer contrast between stained cells and surrounding tissue. Gram+Reg detects the transition from the cortex to white matter better than GAN+Reg. Both methods introduce an artificial arrangement of cells into cortical columns not present in the original Cresyl violet staining.

To further analyze the ability of the methods to reconstruct the laminar cell organization, we compute grey level index (GLI) values (Zilles et al., 1978). GLI values provide an established proxy for volume density of stained cell bodies in gray matter regions and are used to characterize the laminar architecture of cortical areas (Schleicher et al., 2000). To compute GLI values, adaptive thresholding is applied to create a mask of pixels occupied by cell bodies for each image. The masks are subsequently down scaled by a factor of 16 to a spatial resolution of 20.8 µm, with each value representing the fraction of segmented pixels. Resulting GLI images, cropped to the area between pial surface and the cortex/white matter transition, are displayed for the Cresyl violet target and the predictions in Figure 8D.

For each of the GLI images, 31 intensity profiles vertically oriented to the cortical layers are extracted to represent the columnar distribution of GLI values. The profiles are restricted to layers II - VI since staining inhomogeneities make GLI values for layer I unreliable. To obtain one representative profile for each image, the profiles are averaged and smoothed using a mean filter with kernel size 3 to improve the signal-to-noise ratio. The average profiles are shown on top of each GLI image in Figure 8D.

The average GLI profile for the GAN model shows no clear distinction between cortical layers II-IV and misses a peak for layer IV. The profile by Gram replicates peaks for layers II and IV but does not provide a clear representation of the cortical layers. GAN+Reg and Gram+Reg both replicate peaks for the higher cell densities in layers II, IV and VI. Gram+Reg shows the clearest distinction between layers and even replicates slightly nuanced peaks of the profile within layers.

### 3.4 Effect of loss weighting parameters

To incorporate equivariance loss ${ℒ}_{E}$ into previous models, we set its weighting parameter to η=0.1 . To validate this choice, we perform training of Gram+Reg models with η values ranging from 0 to 10. Results in Table 3 indicate that performance is robust for η>0 . Only when the equivariance loss is entirely removed from training (setting η=0 ), we observe a measurable negative effect on the results. We report mean values and standard errors over four independent trainings to show the significance of this effect.

**Table 3.** Ablation study on weighting parameter η of equivariance loss ${ℒ}_{E}$ for a Gram+Reg model.

Method	η	MI ↑	RMSE ↓	SSIM ↑	F1 ↑
Gram+Reg	0.0	0.211 ± 0.004	30.2 ± 0.4	0.432 ± 0.004	35.2 ± 1.2
	0.01	**0.225** ± 0.002	29.5 ± 0.3	**0.446** ± 0.001	**41.6** ± 0.2
	0.1	0.224 ± 0.003	29.6 ± 0.6	0.445 ± 0.003	41.0 ± 0.4
	1.0	0.220 ± 0.001	**29.4** ± 0.1	0.445 ± 0.001	41.4 ± 0.2
	10.0	0.224 ± 0.002	**29.4** ± 0.3	**0.446** ± 0.001	41.2 ± 0.2

Performance is robust across a wide range of η>0 values. Only when the loss term is completely removed (η=0), we observe a measurable degradation in performance. The deviation is reported as standard error over four independent trainings with different random seeds. Arrows indicate the direction of better performance (↑ higher is better, ↓ lower is better). Best scores per column in bold.

To identify an optimal weighting λ of the style loss in Eq. (10), we train Gram+Reg models with different values for λ and MSE online registration metric. A quantitative evaluation of the trained models in Table 4 shows that larger values for λ up to λ = 0.75 achieve the best evaluation scores. This indicates that the reconstruction loss should be given a stronger weight but should not be used exclusively (λ=1 ).

**Table 4.** Different values for weighting parameter λ show that a balance between style loss ${ℒ}_{S}$ and reconstruction loss ${ℒ}_{R}$ is required in the training of a Gram+Reg model.

Method	λ	MI ↑	RMSE ↓	SSIM ↑	F1 ↑
Gram+Reg	0	0.124	33.8	0.340	28.6
	0.03	0.162	31.9	0.394	36.1
	0.25	0.210	30.7	0.436	39.8
	0.5	0.226	**29.8**	0.444	41.3
	0.75	**0.238**	**29.8**	**0.448**	**41.4**
	0.97	0.230	30.7	0.446	40.2
	1.0	0.090	38.0	0.347	0

Setting λ=0 means only ${ℒ}_{S}$ is used. Setting λ=1 means only ${ℒ}_{R}$ is used. Arrows indicate the direction of better performance (↑ higher is better, ↓ lower is better). Best scores per column in bold.

**Fig. 9.**
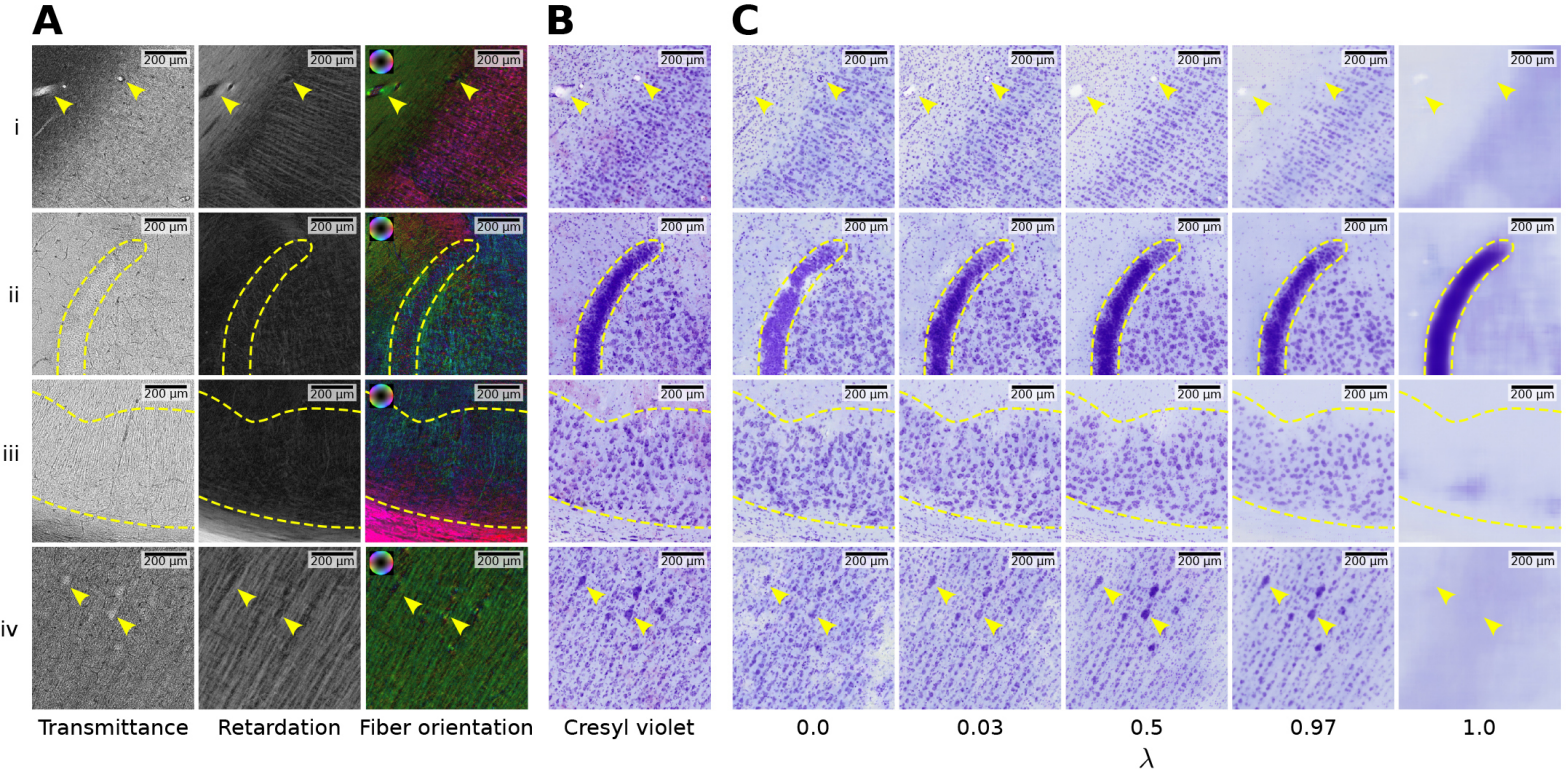
Predicted virtual Cresyl violet stainings for different weightings between reconstruction and style loss components. (A) 3D-PLI inputs as transmittance, retardation and fiber orientation in HSV color space (hue: fiber direction; saturation/brightness: retardation). The retardation has been scaled using gamma correction for visualization. (B) Registered target Cresyl violet staining. (C) Predicted virtual stainings of models trained with different weightings λ between reconstruction loss ${ℒ}_{R}$ and style loss ${ℒ}_{S}$. Setting λ=0 focuses on ${ℒ}_{S}$ and setting λ=1 on ${ℒ}_{R}$ exclusively. Gram loss is used for ${ℒ}_{S}$. Within each row, yellow markers indicate the same structures across all columns. (i) Temporal area TE and underlying white matter with arrowheads highlighting blood vessels within white matter and cortical layer VI. (ii) Dentate gyrus of the hippocampus with a dashed line delineating the proximal end of the granular layer of the fascia dentata (FD). (iii) Pyramidal layer of the cornu Ammonis (CA1) region of the hippocampus highlighted by dashed lines. (iv) Layer V of the primary motor cortex with arrowheads highlighting two Betz cells.

A visual comparison of predictions of Gram+Reg models trained with different choices for λ is shown in Figure 9 for selected crops from the test data. By using ${ℒ}_{ℛ}$ only (i.e., λ=1 ), the model is unable to reconstruct details from the Cresyl violet staining. It is only able to locate strongly pronounced structures such as the granular layer delineated in (ii). Independent of the style loss weighting, the models do not resolve individual cell instances present within the granular layer. Instead, they capture the overall cell density, represented by a continuous dark purple color. By increasing emphasis on ${ℒ}_{S}$ (i.e., with decreasing values for λ), the generator is able to reconstruct details such as neuronal cell bodies in less dense regions in (i)-(iv) and blood vessels in (i). It is also able to generate Glial cells to match the appearance of white matter in (i) and (iii). However, generators trained with an overweighting of style loss also tend to miss some strongly pronounced structures in the predictions, such as the blood vessels shown in (i) or the Betz cells in (iv).

### 3.5 Influencing factors on model predictions

To assess the reliability of our method under varying biological and imaging conditions, we analyze in how far the reconstruction of cell instances depends on cell size and local strength of birefringence. We also examine how different focus levels of the LMP-1 impair the quality of the virtual staining.

**Fig. 10.**
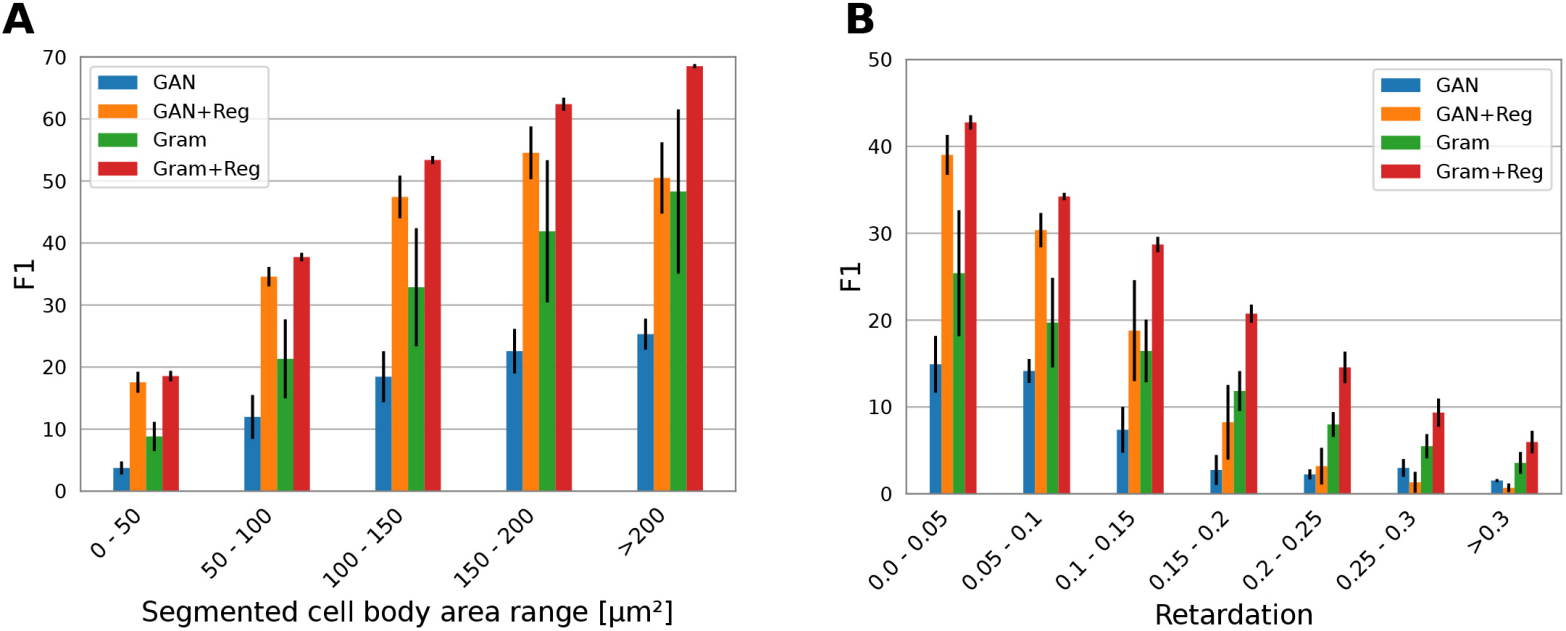
Reliability of predicted cell instances increases with their size and decreases with a stronger retardation. We report F1 scores between detected cell instances by a CPN model in the predicted and target stainings. F1 scores are computed for intervals of (A) the segmented in-plane cell body area and (B) smoothed retardation maps. Retardation values are sampled at each cell location. Each bar represents the average over four independently trained models. Error bars show standard deviation.

Larger cells are expected to be more pronounced in 3D-PLI parameter maps compared to smaller cells that may be overshadowed by other tissue components such as nerve fibers. To quantify the reliability of predicted cells in the virtual staining in relation to their size, we compute F1 scores for multiple bins of 2D in-plane cell sizes. Obtaining F1 scores for each bin requires computation of true and false positives, as well as false negatives through matching of predicted and target cells with a minimum IoU threshold of 0.3. We modify the computation of true and false positives by matching only predicted cells within that range with all cells in the target image. To count false negatives, target cells within that range are matched with all cells in the prediction. Results in Figure 10A show that reconstruction of smaller cells < 50 µm² has much lower F1 scores, below 20.0, throughout all methods. With increasing 2D cell size, they can be identified more accurately. The Gram+Reg model shows highest F1 scores across all cell sizes and smallest variation between four independently trained models, measured as standard deviation.

Birefringent tissue, such as myelinated nerve fibers, can obscure signals from other components in 3D-PLI. To examine whether this effect impacts the prediction of cell instances in the virtual staining, we use retardation maps as a measure of birefringence strength. The maps are smoothed with a 10 pixels square median kernel to reduce local variance and obtain values representative of the surrounding tissue area. For each cell instance segmented by the CPN model in the virtual staining, we sample a retardation value at its center location. Values are grouped into intervals from 0.0 to 0.3 in steps of 0.05. Since we focus the analysis on gray matter, larger retardation values do not occur. As shown in Figure 10B, the ability of all models to reconstruct cell instances decreases significantly for cells dominated by stronger retardation signals. For Gram+Reg this effect is minimal, performing overall best.

**Fig. 11.**
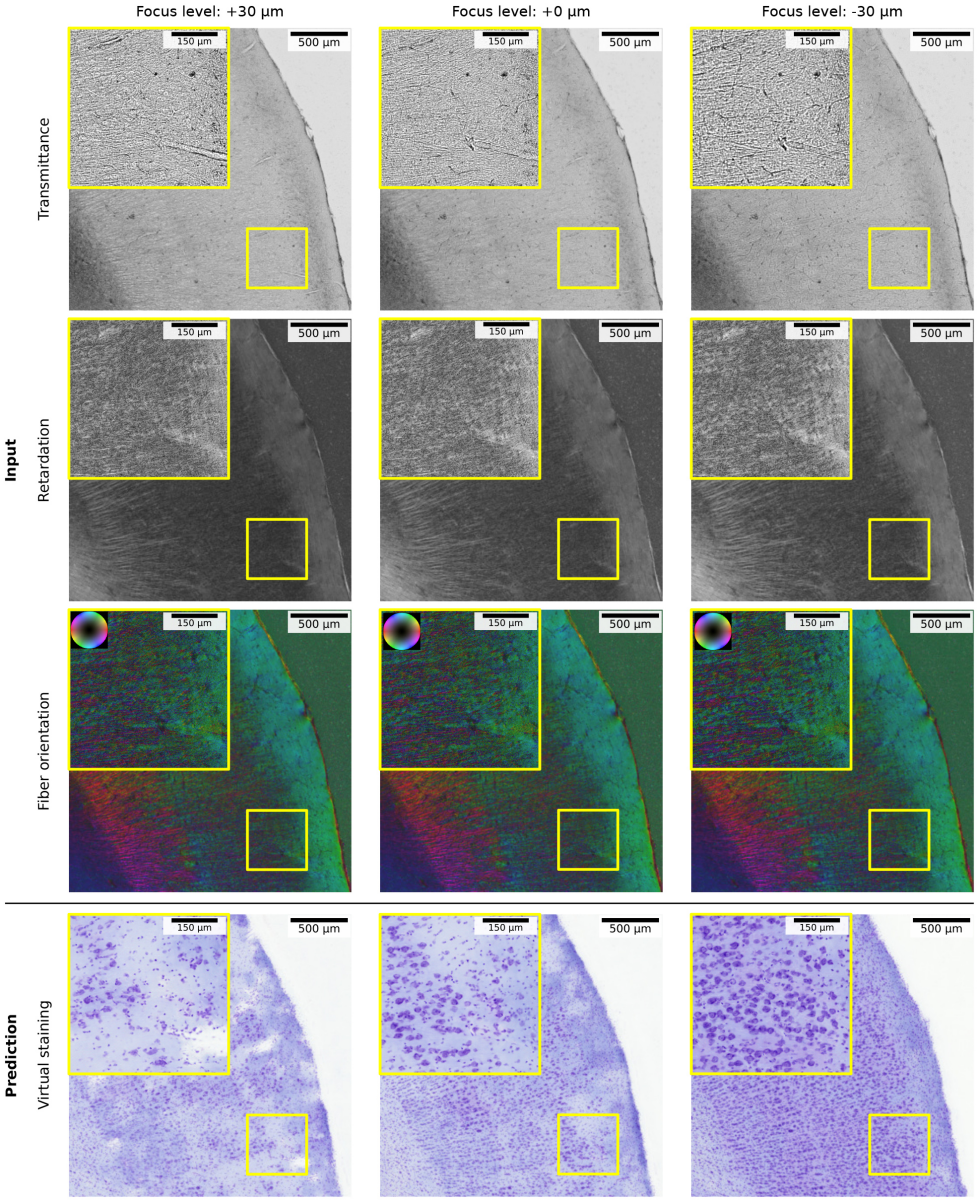
Virtual staining quality varies with the focus level of the LMP-1 microscope, which influences the captured diffraction patterns. Example images show the vervet entorhinal cortex with zoom-ins to the architecture of cortical layers II-III. 3D-PLI inputs are represented as transmittance, retardation (scaled with gamma correction) and fiber orientation in HSV color space (hue: fiber direction; saturation/brightness: retardation). The same Gram+Reg model is used to predict virtual stainings from a focus level adjusted to the tissue center (+0 µm) and 30 µm above and below.

We observe that the virtual staining intensities are not always homogenous within generated images. Such effects can especially be observed as cloud-like patterns in Figure 5 for the predicted virtual staining of ROIs showing the primary motor cortex and subcortical nuclei. These variations coincide with variations in the diffraction pattern of the associated transmittance maps, which in turn can be influenced by the focus level of the LMP-1 microscope. To better quantify this relationship, we apply a Gram+Reg model to predict virtual stainings from an example image showing the vervet entorhinal cortex, captured at three focus levels: centered on the tissue (+0 µm), -30 µm and +30 µm. Results are summarized in Figure 11. We observe the highest consistency in staining intensity at the lowest focus level of (-30 µm). Higher focus levels introduce an increasing amount of staining artifacts.

## 4 Discussion

Our ablation experiments in Sections 3.2, 3.3 and 3.4 support the design decisions of the proposed virtual staining method. In Section 3.5, we investigated several influencing factors on the predictions. In the following, we want to discuss the design decisions of the proposed virtual staining method and assess its reliability for downstream analysis.

We introduced an online registration head capable of approximating smooth nonlinear deformations, given sufficiently small patch sizes for training. Its accuracy is inherently influenced by the choice of the registration metric. We identified MSE in the Fourier domain through multiple applications of cross-correlation as superior to other typically used metrics in Fourier-based image registration, including PC. This is a notable observation, as PC is a popular choice due to its robustness to intensity variations and frequency-dependent noise (Tong et al., 2019). However, during early training stages, model predictions are often blurred. The network tends to learn coarse, low-frequency structures first. Additionally, random weight initialization introduces noise in the first steps. MSE appears more resilient to such blur and frequency-independent noise, leading to more accurate predictions (Fig. 6). This behavior can be explained by PC’s reliance on strong high-frequency spectral peaks, which are diminished by blur (Pedone et al., 2013). MSE, in contrast, maintains sensitivity across the frequency spectrum and can better handle smooth, low-detail inputs that dominate during initial training.

The applied pixel-wise reconstruction loss assumes a precise alignment to provide an informative feedback signal. However, the online registration head will typically only succeed if a good contrast between structures is being predicted. In other words, there is a cross-dependency of accurate pixel reconstructions and producing a high contrast over training iterations, which may result in a conflict during training. This can be seen in Figure 9, where a Gram+Reg model focussing on pixel reconstruction only made heavily blurred predictions that do not provide structural details for cell-precise registration. Adding a style loss component leads to more pronounced contours while simultaneously achieving higher pixel reconstruction accuracy (Table 4) through online registration. This underpins the importance of the additional style loss, which does not depend on perfect alignment and guides the training to produce structures. Still, some manual pre-alignment was required to keep the registration error of training data within 70 pixels (91 µm) and ensure sufficient structural overlap between training patches. This limited the amount of available training data, as larger registration errors, such as those from the initial affine alignment of whole sections, were too large for the online registration to succeed.

Apparently, the choice for Gram or GAN as style loss should be considered in the light of the application. Training with Gram loss led to more accurate cell localization and pixel values (Table 2). However, it was affected by staining intensity inhomogeneities, resulting in blurry cells (Fig. 8). GAN loss, on the other hand, produced higher contrast in cells but with lower accuracy. This could be a consequence of the Gram loss referring to the specific target image, while the GAN loss refers to the average scoring of the discriminator across the distribution, less than the precision with respect to an individual image.

The choice of style loss function also impacts the stability and complexity of the training process. When using GAN loss, the training process has to be carefully monitored. If training collapses, restarting is often necessary. It requires careful balancing of discriminator and generator capacities and tuning of style and reconstruction loss weighting. Training with Gram loss, on the other hand, is generally more convenient to train. It requires no balancing of model capacities, is robust to loss balancing, and does not collapse during training.

We expect that the expressiveness of the Gram matrix representation could be enhanced by replacing the VGG encoder, pre-trained on ImageNet, with a domain-specific model, which is powerful on microscopy data. This is technically motivated by the observation that higher-level features in ImageNet-pretrained networks, such as VGG, are optimized for object semantics in natural images (e.g., faces, animals, vehicles), which are irrelevant in the context of fine-grained texture characteristic of microscopy images (Stuckner et al., 2022). In contrast, microscopy-specific encoders are better suited to capture such low- to mid-level textural features and have been shown to improve clustering (Oberstrass et al., 2024), segmentation (Stuckner et al., 2022), and the evaluation of generative models (Kropp et al., 2024) over those trained on ImageNet. Therefore, computation of Gram matrices using domain-specific encoders (Schiffer, Amunts, et al., 2021; Schiffer, Spitzer, et al., 2021; Spitzer et al., 2017) or emerging foundation models for histology (Tran et al., 2024) might further enhance representation of cytoarchitectural characteristics.

Throughout all models, we observed occasional staining inhomogeneities in the predicted virtual stainings. An example of local-scale variation is the inhomogeneity observed in Figure 8, which may be attributed to the tiling strategy used during model inference to transfer whole sections. Since the model lacks a global view of the section, it may reproduce variations in the training data at the local patch level. At a larger scale, this effect was pronounced in the form of stripe patterns, for example in the primary motor cortex ROI (Fig. 5). These patterns typically coincide with inhomogeneities in the 3D-PLI transmittance maps. During the mounting process of the 60 µm thick sections in 20% glycerol solution on the object slide the gray matter is more prone to swelling than the more compact white matter, leading to tissue expansion, mechanical stress and tape flutter especially in the cortex due to its higher flexibility. The free-floating tissue between object slide and coverslip shapes waves of up to 30 µm in amplitude. These waves affect the focus level and the light incidence on the tissue, hence the effective birefringence, modifying the polarization state of the transmitted light. This caused the observed stripe patterns in the transmittance maps and very likely contributed to the staining inhomogeneities.

Cell body membranes and blood vessel walls pose hard edges in the cortex, resulting in diffraction patterns even brighter than the background field. Varying of the focus level of the LMP-1 microscope allows optical scanning of the Poisson spots induced by diffraction. Cell bodies are highlighted with higher contrast by shifting and lowering the focal plane to the top and the bottom of the tissue by approximately ±30 µm (Fig. 11). The ability to predict cells from 3D-PLI measurements was shown to be sensitive to variations in the focus level. An effective countermeasure against waves in the tissue, to maintain a constant level across the entire section, is to weight the open coverslips after embedding overnight. In addition, the acquisition of multiple levels for each section could potentially provide missing information. For the given dataset, however, these potential solutions were not applicable, and measurements could not be easily repeated. Since shifting the focus toward the tissue surface degrades optical resolution, a center focus level is used by default in 3D-PLI for an optimal representation of nerve fibers. The acquisition of multiple focus levels of ±30 µm to increase the visibility of cell bodies would significantly increase the measurement effort. Nevertheless, it will be an important consideration for future investigations to include multiple focus levels in each measurement to improve cell extraction.

It must be noted that shrinking and swelling of the tissue also affects the measured areas of individual 2D segmented cell bodies. Tissue deformation in histology is complex, involving anisotropic effects and differences between cellular and extracellular structures (Dorph-Petersen et al., 2001; West, 2013), which cannot be fully captured by the global shrinkage factor estimated in Section 2.1.3. At the same time, there are only sparse data available that quantifies such effects, and the precise conditions of histological processing differ between each other, and it can be assumed that such differences influence the specific shrinkage values. In paraffin-embedded human sections, a mean difference of 9% between gray and white matter shrinkage has been reported (Kretschmann et al., 1982). As frozen sections generally show less in-plane deformation than paraffin sections (West, 2013), we treat this value as an upper bound for our acqusition. Since gray matter contains relatively more cell bodies than white matter, similar shrinkage of gray and white matter suggests that shrinkage of cell bodies and neuropil should also not differ significantly (Amunts et al., 1999). By performing non-linear co-registration of Cresyl violet to 3D-PLI and applying a subsequent global correction of cell sizes to account for swelling effects from MRI to 3D-PLI, we assume that major shrinkage effects are compensated. However, residual local variations or relative slice-to-slice differences may persist and will require a more systematic approach in future work.

While most of the larger cells (>100 µm² in-plane size) could be localized in 3D-PLI parameter maps, smaller cells were often missed or misplaced in the predictions. This is expected as smaller cell structures are often dominated by a stronger signal of intersecting nerve fibers or overlay each other due to the high section thickness of 60 µm required for 3D-PLI. In our experiments, we observed that stronger birefringence, corresponding to higher retardation values, resulted in reduced capability of models to reconstruct cell instances. Since birefringence increases with myelin density and homogeneity, predictions in regions with densely packed myelinated nerve fibers, such as white matter, are thus less reliably captured by the virtual stainings. In white matter, detecting cell bodies may even be infeasible, as the predominant oligodendrocytes form the myelin sheath and are therefore indistinguishable from it. In gray matter, this limitation may explain the stronger arrangement of cells into cortical columns in the predictions compared to the target images in Figure 8, as the strong signal of myelinated radial fibers can obscure underlying cell instances. The presence or absence of this arrangement is one of the criteria used to identify cortical areas, which might impair downstream interpretation of the predicted architecture.

As shown by the differences in F1 values in Appendix Table A1, cells were reconstructed more accurately in isocortical areas (motor cortex and temporal cortex) than in the hippocampus or the subcortical nuclei (putamen and globus pallidus). The lower F1 in the putamen compared to the motor and temporal areas can be attributed to its neuronal composition and the fundamentally different spatial arrangement of cell bodies in cortical versus subcortical regions, as well as their relation to fiber bundles. The putamen is composed of medium-sized spiny neurons and relatively small interneurons arranged around and between bundles of myelinated fibers, which course through the putamen and give rise to its characteristic striated appearance (Heilbronner et al., 2025). In this region, two challenges reduce predictability: small interneurons are difficult to detect with our approach, and fiber bundles may intersect or obscure cell bodies, making them harder to reconstruct. Moreover, the cell bodies of spiny neurons can resemble fiber bundles oriented perpendicular to the section plane, appearing as dark patches in the transmittance image. The globus pallidus contains relatively large but sparsely distributed neurons, which are embedded in a dense matrix of multidirectionally oriented myelinated fibers (Heilbronner et al., 2025). Here, predictability is reduced by the number and complex spatial organization of intersecting myelinated fiber bundles and blood vessels. The low F1 value in patches extracted from the CA1-CA3 regions of the hippocampus is more difficult to explain, though it may result from the fact that they cover both the pyramidal layer, with its numerous and relatively large pyramidal cells, and the radiatum layer, which contains sparsely packed and relatively small interneurons (Zhao & Palomero-Gallagher, 2025), which are difficult to detect with our method.

Our Gram+Reg model produced larger cells at expected positions and generated images with a plausible appearance, making virtual stainings useful for cross-modality registration - at present the most relevant application. They also allowed the application of cytoarchitectonic tools such as cell segmentation or computation of GLI profiles for cortical layer characterization on 3D-PLI images. As a future perspective, the ability to detect cell bodies in 3D-PLI may enhance the computation of 3D fiber orientations within gray matter, which is a prerequisite for fiber tractography. This could be achieved by improving the estimation of myelination, for example through the identification of voxels dominated by cell bodies, and potentially also support the localization of axon terminals. However, virtual stainings occasionally contained artifacts, including staining inhomogeneities, omission of smaller cells, or the introduction of implausible cytoarchitectonic features. These issues may reflect biases in the training data, model-related artifacts, or missing cellular signatures in the 3D-PLI parameter maps. The virtual staining allowed the identification by a neuroanatomist (N.P.-G.) of the borders between the CA1, CA2, and CA3 regions of the hippocampus, the border between the primary motor and primary somatosensory areas, or the border between the retrosplenial cortex and cingulate area 23. However, it was not possible to identify the border between the core and lateral belt auditory areas, because it is characterized by differences in the packing density of small pyramids (Hackett et al., 2001), which currently cannot be reliably predicted. Therefore, we do not yet consider the method sufficiently robust for reliable cytoarchitectonic brain area mapping. Nevertheless, we are convinced that expanding the number of training sections, brain regions, and focus levels across brains and species can improve model performance and generalizability. While such expansion would require re-training, each additional sample would teach the model novel architectural patterns, improving its robustness across domains.

## 5 Conclusions

Motivated by previous observations that larger cells are encoded in 3D-PLI parameter maps alongside fiber orientations (Zeineh et al., 2017), we introduced a deep learning model for transforming 3D-PLI maps into virtual Cresyl violet cell body stainings. This approach enables joint visualization of fiber tracts and cell bodies in the same tissue. Compared to real post-staining, the model may offer a scalable alternative that avoids manual labor.

A central contribution of our approach is the integration of an online registration head during training. This component eliminates the need for explicit, pixel-accurate multimodal registration, which is commonly required in virtual staining pipelines (de Haan et al., 2021; Rivenson et al., 2020; Yang et al., 2022). It is a simple but highly efficient add-on that can be combined with various loss formulations, leveraging model-estimated landmarks, to continuously refine the alignment over time.

The developed method enables localization of most of the larger cell bodies in gray matter (>100 µm² in-plane size) from 3D-PLI and a successful adaptation to the appearance of real Cresyl violet stainings. As such, it expands the usability of 3D-PLI in large-scale data settings, allowing virtual staining at scale. While such synthetic data cannot and should not replace real histological measurements, it offers promising opportunities in downstream analysis. Although we see the present method not yet robust enough for cytoarchitectonic mapping, potential applications include cross-modal image registration to align real Cresyl violet and 3D-PLI, performing cell segmentation in 3D-PLI images, or missing data imputation in serial section stacks. Especially in interleaved modalities, this could enable the reconstruction of complete datasets. Of course, such applications require careful quality control, a clear separation and demarcation of synthetic data from real measurements, and careful interpretation of derived results.

The outcomes of this study lay the groundwork for prospective investigations focused on enhancing 3D-PLI analysis, particularly through the exploration of dedicated cell detection techniques to directly extract cell body instances from 3D-PLI data. Furthermore, the findings serve as a motivation for gathering additional training data, aiming to refine and extend the application of the virtual Cresyl violet staining to a broader range of sections, brains, and species. While the current model is trained specifically to replicate Cresyl violet stainings, the same methodology can in principle be adapted to other staining types, provided appropriate retraining is performed. Future research should focus on further investigating its transferability to other datasets, staining protocols, and brains.

## Data Availability

The training pipeline for presented Gram+Reg and GAN+Reg models is available on GitHub.^4^ Code for the online registration,^5^ data augmentations for 3D-PLI images,^6^ visualization methods for 3D-PLI modalities,^7^ as well as additional dependencies^8^ are hosted on our external GitLab server. ROIs employed for the training and testing of the models in this study, along with a selection of model predictions, are available in our central institutional repository (Oberstrass et al., 2025). In addition, the repository includes whole-slide predictions for test section 559 along with corresponding 3D-PLI modalities.

## Footnotes

1
https://github.com/HelmholtzAI-Consultants-Munich/Quicksetup-ai

2 ginoro_CpnResNeXt101UNet-fbe875f1a3e5ce2c

3
https://github.com/FZJ-INM1-BDA/celldetection

## Appendix

**Appendix Table A1.** Specific quantitative results per test ROI.

ROI	Method	MI ↑	RMSE ↓	SSIM ↑	F1 ↑
Motor cortex	Gram	0.061 ± 0.013	33.9 ± 1.0	0.306 ± 0.024	24.8 ± 3.5
	Gram+Reg	**0.168** ± 0.003	**29.2** ± 0.6	**0.446** ± 0.002	**42.9** ± 0.3
	GAN	0.117 ± 0.016	34.7 ± 1.1	0.268 ± 0.010	16.8 ± 1.0
	GAN+Reg	0.165 ± 0.015	29.6 ± 1.0	0.394 ± 0.013	36.3 ± 2.1
Hippocampus	Gram	0.184 ± 0.015	34.5 ± 0.9	0.340 ± 0.013	19.4 ± 2.3
	Gram+Reg	**0.303** ± 0.003	**31.1** ± 0.5	**0.448** ± 0.001	**31.4** ± 0.4
	GAN	0.078 ± 0.016	44.3 ± 3.1	0.262 ± 0.016	6.9 ± 1.3
	GAN+Reg	0.151 ± 0.036	32.1 ± 0.6	0.373 ± 0.027	24.9 ± 1.9
Temporal cortex	Gram	0.077 ± 0.018	33.9 ± 0.8	0.319 ± 0.040	31.7 ± 4.5
	Gram+Reg	**0.236** ± 0.003	**29.1** ± 0.4	**0.497** ± 0.004	**52.7** ± 0.4
	GAN	0.051 ± 0.006	43.1 ± 5.4	0.238 ± 0.019	18.3 ± 2.7
	GAN+Reg	0.192 ± 0.015	30.1 ± 1.5	0.460 ± 0.017	49.6 ± 1.3
Subcortical nuclei	Gram	0.122 ± 0.007	32.0 ± 0.6	0.290 ± 0.016	15.7 ± 2.2
	Gram+Reg	**0.190** ± 0.004	**29.2** ± 0.4	**0.388** ± 0.001	**29.4** ± 0.5
	GAN	0.138 ± 0.028	33.0 ± 0.8	0.245 ± 0.013	11.4 ± 0.9
	GAN+Reg	0.132 ± 0.014	29.9 ± 0.7	0.330 ± 0.014	20.9 ± 1.6

The deviation is reported as standard error over four independent trainings with different random seeds. Arrows indicate the direction of better performance (↑ higher is better, ↓ lower is better). Best scores per ROI in bold.
